# Near real-time data on the human neutralizing antibody landscape to influenza virus in summer of 2026 shows antigenic advance of H3N2 subclade K region D mutants and H1N1 D.3.1.1 Sa mutants

**DOI:** 10.64898/2026.09.15.751855

**Published:** 2026-09-20

**Authors:** Caroline Kikawa, Andrew Butler, John Huddleston, Sam A. Turner, Heidi Peck, Janet A. Englund, Kirsten Lacombe, Michael Busch, Marion C. Lanteri, Mars Stone, Bryan Spencer, Alexander L. Greninger, Derek J. Smith, Stephanie Wallace, Helen S. Marshall, Shidan Tosif, Scott E. Hensley, Ian G. Barr, Jesse D. Bloom

**Affiliations:** 1 Division of Basic Sciences and Computational Biology Program, Fred Hutch Cancer Center, 1100 Fairview Ave N, Seattle, WA 98109, USA; 2 Department of Genome Sciences, University of Washington, 1959 NE Pacific St, Seattle, WA 98195, USA; 3 Medical Scientist Training Program, University of Washington, 1959 NE Pacific St, Seattle, WA 98195, USA; 4 Vaccine and Infectious Disease Division, Fred Hutch Cancer Center, 1100 Fairview Ave N, Seattle, WA 98109, USA; 5 Center for Pathogen Evolution, Department of Zoology, University of Cambridge, The Old Schools, Trinity Ln, Cambridge CB2 1TN, United Kingdom; 6 WHO Collaborating Centre for Reference and Research on Influenza, The Peter Doherty Institute for Infection and Immunity, 792 Elizabeth Street Melbourne, 3000, Australia; 7 Seattle Children’s Research Institute and Department of Pediatrics, University of Washington, 1959 NE Pacific St, Seattle, WA 98195, USA; 8 Vitalant Research Institute, 360 Spear St Ste 200, San Francisco, CA 94105, USA; 9 Department of Laboratory Medicine, University of California San Francisco, 505 Parnassus Avenue, San Francisco, CA 94143, USA; 10 Creative Testing Solutions, 2424 W Erie Dr, Tempe, AZ 85282, USA; 11 American Red Cross, 180 Rustcraft Rd, Dedham, MA 02026, USA; 12 Department of Laboratory Medicine and Pathology, University of Washington Medical Center, 1959 NE Pacific St, Seattle, WA 98195, USA; 13 University of the Sunshine Coast Clinical Trials, Sunshine Coast, Queensland, Australia; 14 Adelaide University, South Australia, 5005, Australia; 15 Murdoch Children’s Research Institute, University of Melbourne, The Royal Children’s Hospital Melbourne, 50 Flemington Road, Parkville, Victoria, 3052, Australia; 16 Depts of Microbiology and Medicine, Perelman School of Medicine, University of Pennsylvania, 3451 Walnut Street, Philadelphia, PA 19104, USA; 17 Howard Hughes Medical Institute, 1100 Fairview Ave N, Seattle, WA 98109, USA

## Abstract

Human seasonal influenza evolves rapidly, necessitating twice yearly decisions about whether to update the strains in the vaccine. To help inform this decision, we have been using high-throughput sequencing-based neutralization assays to make twice yearly measurements of how recent human sera neutralize current human H3N2 and H1N1 strains. Here we provide the third installment in this series of measurements by reporting 52,268 titers representing neutralization of 148 viral strains by 355 human sera collected between April and August of 2026. Our measurements show that new H3N2 subclade K strains with mutations in antigenic region D and new H1N1 subclade D.3.1.1 strains with mutations in antigenic region Sa (such as G155E) have reduced neutralization by human sera, with notable heterogeneity in the impact of some of these mutations across sera from different individuals. This paper is accompanied by an interactive summary (https://jbloomlab.github.io/flu-seqneut-2026/summary.html) that enables detailed exploration of the results, and all titer data are publicly available for further analysis to aid vaccine antigen selection and studies of viral evolution.

## Introduction

The hemagglutinin (HA) protein of human seasonal influenza evolves rapidly to erode neutralizing antibodies (Smith et al. 2004; Bedford et al. 2014). To keep pace with this evolution, the influenza vaccine is updated regularly, with a decision made twice per year about whether to change the strains in the vaccine to better match circulating strains. Because it takes time to produce and administer the vaccines, the strain update decision is made roughly seven to ten months before the influenza season during which the vaccine will be deployed: vaccine strains are chosen in September for the following year’s Southern Hemisphere influenza season, and in February for the Northern Hemisphere influenza season that begins at the end of the same year (World Health Organization 2026; World Health Organization 2025). This gap between the timing of the vaccine update decision and the influenza season requires forecasting which strains will dominate nearly a year in the future.

The influenza strains that spread in the human population tend to have mutations in HA that reduce neutralization by pre-existing antibody immunity (Bedford et al. 2015; Smith et al. 2004; Łuksza and Lässig 2014; Huddleston et al. 2020; Neher et al. 2016; Kikawa et al. 2026b); such mutations increase viral fitness by making a larger fraction of the human population susceptible to infection (Kim et al. 2024; Petrie et al. 2016; Hobson et al. 1972). Therefore, identifying strains and mutations that reduce antibody neutralization is important for vaccine-strain selection. However, viral fitness also depends on inherent transmissibility (which can be affected by mutations in multiple gene segments) (Raghwani et al. 2017; Liu et al. 2024), pre-existing antibody immunity to the other surface protein neuraminidase (Krammer 2019; Monto et al. 2015), and to a lesser extent pre-existing T-cell immunity (Machkovech et al. 2015; Boon et al. 2002). Therefore, measuring how the HAs of different viral strains are neutralized by human antibodies is an important component of viral forecasting—but the strains with the lowest neutralizing titers may not always be the most successful ones.

The last year of evolution of seasonal H3N2 and H1N1 influenza has seen the spread of new viral subclades with reduced neutralization by human HA-directed neutralizing antibodies. In the late summer of 2025, subclades K and D.3.1.1 arose within H3N2 and H1N1 subtypes, respectively, and rapidly increased in frequency. Subclade K has six HA1 mutations relative to its parent subclade J.2.4, mostly in antigenic regions A and B (Liu et al. 2026b). Subclade D.3.1.1 has four HA1 mutations relative to parent subclade D.3.1. These subclades were antigenically advanced relative to parent subclades, with most studies measuring a 1.5- to 2-fold decrease in neutralization by human sera of subclade K (Kikawa et al. 2026a; Kirsebom et al. 2025; Cheng et al. 2026; Dee et al. 2026; Ikonen et al. 2026; Separovic et al. 2026; Wang et al. 2026; Liu et al. 2026b) and a smaller but measurable decrease in neutralization of D.3.1.1 (Kikawa et al. 2026a). Descendant variants of both subclade K and D.3.1.1 are now spreading in the human population, underscoring the continuing need to rapidly measure the antigenic properties of new influenza strains.

Sequencing-based neutralization assays can measure the titers of many sera against many viral strains (Kikawa et al. 2026b; Loes et al. 2024). These assays are higher throughput than conventional neutralization or hemagglutination-inhibition assays because they use a sequencing-based readout to measure each serum’s neutralization of >100 viral strains at once. In September of 2025, we started using sequencing-based neutralization assays to make near real-time measurements of the human neutralizing antibody landscape against the current diversity of seasonal H3N2 and H1N1 influenza strains, with these measurements timed to help inform vaccine update decisions (Kikawa et al. 2025; Kikawa et al. 2026a). Here we continue this effort by measuring the neutralization of 148 human H3N2 or H1N1 seasonal strains by 355 recently collected human sera, providing 52,268 total titer measurements that identify new naturally emerging viral strains with HA mutations that reduce recognition by human neutralizing antibody immunity.

## Results

### A library of naturally occurring H3N2 and H1N1 HAs that cover the current diversity of human seasonal influenza

For neutralization titer measurements to be maximally useful for vaccine-strain update decisions, the influenza viruses being assayed should be representative of the current diversity immediately preceding this decision. Towards this end, we designed a library of influenza HAs with the goal of covering the diversity of H3N2 and H1N1 HAs circulating in humans leading up to the September 2026 vaccine-composition meeting.

**Figure 1.**
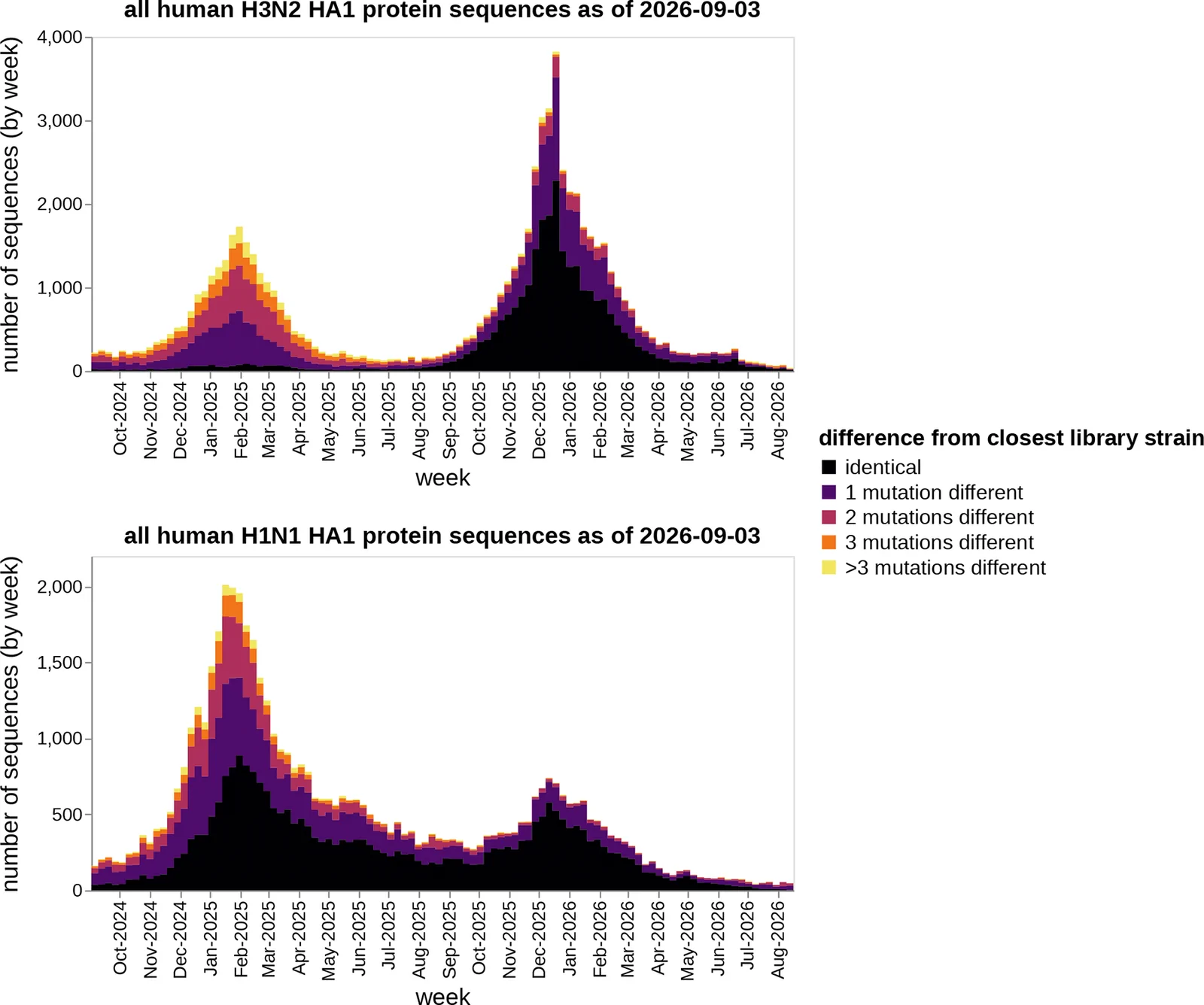
The sequencing-based neutralization assay library largely covers the HA1 diversity of human seasonal H3N2 and H1N1 influenza over the last year. Number of human seasonal H3N2 (top) or H1N1 (bottom) HA sequences in GISAID (as of September 3, 2026) binned by collection week and colored by HA1 amino-acid identity to the closest strain in our sequencing-based neutralization assay library.

In late May 2026, we surveyed all human seasonal HAs available in the GenBank or GISAID (Shu and Mc-Cauley 2017) databases at that time, and used a combination of computational analysis and manual expert curation to choose a set of HAs from naturally occurring strains based on the following criteria: high overall frequency, rapid increase in frequency over the last six months (Abousamra et al. 2024), mutations at previously established antigenic regions or near/in the receptor binding region (Koel et al. 2013; Wolf et al. 2006; Caton et al. 1982), mutations arising more recurrently than expected by chance (Bloom and Neher 2023; Turner et al. 2026), and observation over a wide geographic range. Overall, this process yielded a set of 77 recently circulating H3N2 HAs and 63 recently circulating H1N1 HAs. We augmented this set with recent egg- and cell-based vaccine strains, for an additional six H1N1 HAs and eight H3N2 HAs. Of the 154 strains we designed for inclusion in the library, 148 could be experimentally generated at sufficient titer, yielding an overall library of 82 H3N2 and 66 H1N1 strains (Supplementary File 1). This library largely covers the diversity of recently sequenced human H3N2 and H1N1 influenza, with the HA1 protein sequence of most sequenced human strains over the last year either identical or within one amino-acid mutation of a strain in our library (Figure 1).

To physically construct this virus library, we individually generated barcoded viruses expressing each HA (Kikawa et al. 2026b; Loes et al. 2024). These viruses contain barcoded HAs with ectodomain protein sequences identical to recently circulating human strains, and all other gene segments from the lab-adapted A/WSN/1933 strain. The viruses were then pooled with the goal of representing all strains at roughly equal titers of transcriptionally active particles, with the pooling done by combining all strains at equal volume, infecting cells, using barcode deep sequencing to quantify the transcriptional titer of each strain, and then re-pooling to balance these titers.

### A set of recently collected human sera

We assembled 355 human sera collected from 260 unique individuals between April and August of 2026 (Table 1 and Supplementary File 2). We included sera from individuals who spanned a wide range of ages (0.5 to 82 years), as past influenza exposure history (which correlates with age) is known to create strong immune imprinting that can affect strain-specific neutralizing titers (Cobey and Hensley 2017). The sera were from three cohorts in the United States, as well as vaccination cohorts in Australia.

The sera from the United States included remnant pediatric sera from hospital visits (mostly unrelated to viral infections) to Seattle Children’s Hospital (SCH cohort), remnant adult sera from routine hepatitis B surface antibody testing at the University of Washington Medical Center in Seattle (UWMC cohort), and sera from blood donors across the United States from Creative Testing Solutions (CTS cohort).

The sera from Australia were provided by the Victorian Infectious Diseases Reference Laboratory (VIDRL), and were collected from children, adults, and elderly individuals. These individuals were sampled pre- and post-vaccination with a 2026 Southern Hemisphere cell-based (Flucelvax) or egg-based (Fluzone or Fluad) seasonal influenza vaccine, which contain a J.2.4 H3N2 strain and a D.3.1 H1N1 strain for the cell-based vaccine, and a J.2.4:F195Y strain and D.3.1:Q223R strain for the egg-based vaccine. As detailed in Table 1, there are differences between the groups that received the different vaccines in terms of post-vaccination sampling time and prior vaccination history.

### Some H3N2 subclade K variants have reduced neutralization, especially those with mutations in antigenic region D

**Figure 2.**
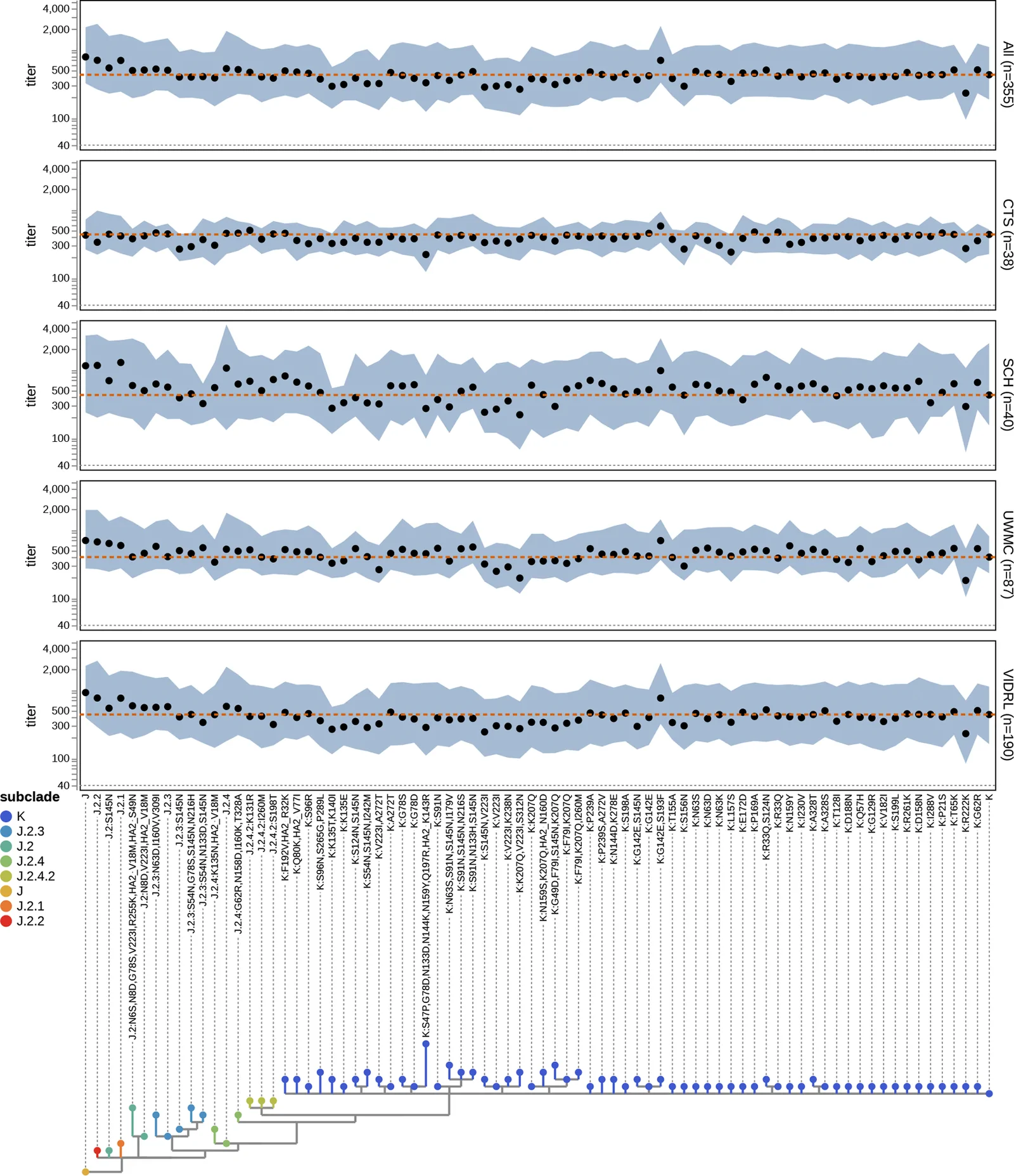
Neutralization titers of all tested sera against recent H3N2 HAs. Points indicate median titer and shaded regions indicate interquartile range. The dashed orange line indicates the titer against subclade K (the 2026–2027 cell-based vaccine strain) and the dotted gray line indicates the lower limit of detection (a titer of 40). Labels to the right of each plot indicate the sera set and number of sera in that set. X-axis labels indicate the HA protein haplotype (subclade and any derived mutations), and the phylogenetic tree shows the relationships among the protein sequences and is colored by subclade.

The neutralization titers against recent H3N2 strains are shown in Figure 2 and reported in Supplementary File 3; see also https://jbloomlab.github.io/flu-seqneut-2026/summary.html for interactive plots that are easier to explore than the static figures in this manuscript.

Across all sera, the lowest H3N2 titers are to subclade K and K-descendant strains. Strains from other recent subclades (J, J.2, J.2.1, J.2.2, J.2.3, and J.2.4) are neutralized by recent human sera similarly or more potently than the parental subclade K strain that is the H3N2 component of the 2026–2027 Northern Hemisphere vaccine (these strains are mostly at or above the orange dashed line in Figure 2). Notably, these other non-K H3N2 subclades have greatly decreased in frequency in the human population over the last nine months, coincident with the rapid spread of subclade K.

**Figure 3.**
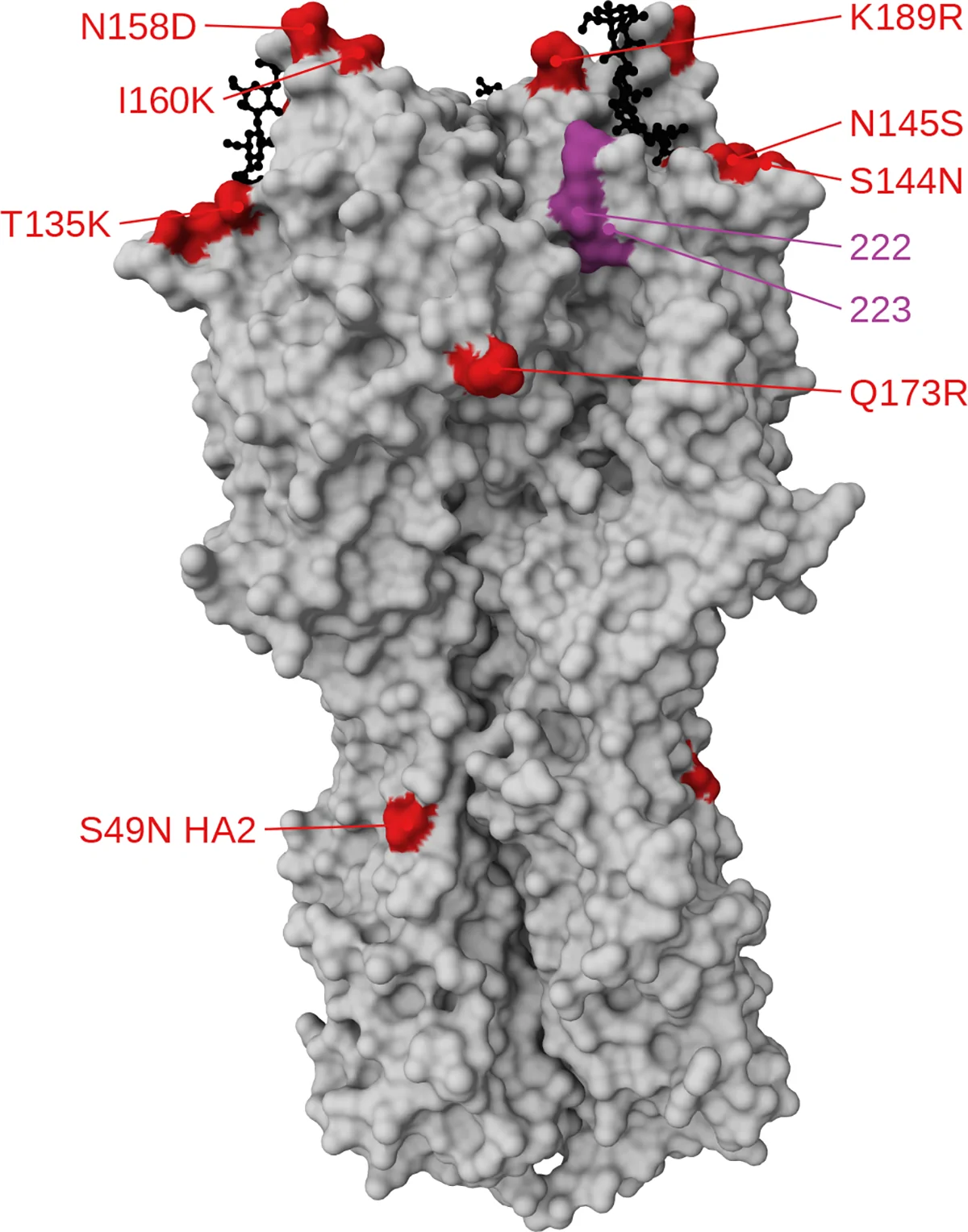
Structure of H3 HA indicating sites 222 and 223 and mutations that separate the 2025–2026 cell-based vaccine (J.2:S145N) and subclade K. Structure of H3 trimer (PDB 8faw) with red indicating mutations that separate the 2025–2026 cell-based vaccine from subclade K, and purple indicating sites 222 and 223 where mutations to subclade K further reduce neutralization. A sialic-acid receptor analogue is shown in black sticks. Sites 135, 144, and 145 are in antigenic region A; sites 158, 160, and 189 are in antigenic region B; sites 222 and 223 are in antigenic region D.

Within subclade K, there are new strains with additional HA mutations that are neutralized substantially less well than the parental subclade K (below the orange dashed line in Figure 2). The subclade K strains with the lowest neutralization mostly carry mutations at HA sites 223 or 222 (e.g., V223I or R222K). Both these sites are in antigenic region D, which was not mutated in the parental subclade K strain—which instead had mutations in antigenic regions A and B relative to its parent subclade J.2.4 (Figure 3). Therefore, mutations in antigenic region D may impair recognition by the antibodies that could still cross-neutralize both subclade K and older strains (Liu et al. 2026a; Kikawa et al. 2026a). Notably, H3N2 HA had an identity of I223 from ~2010 until the emergence of subclade J in ~2022, so some individuals may have antibodies from prior exposures that target I223 variants although overall we still observe V223I to typically cause a decrease in neutralization in subclade K. A mutation at site 222 has only fixed once in human H3N2 evolution (W222R in ~2000). Subclade K strains with mutations at other antigenic region A and B sites (e.g., 145, 156, 157) also had reduced neutralization by some sera relative to the parental subclade K (Figure 2). See https://nextstrain.org/community/jbloomlab/flu-seqneut-2026@main/H3N2 for an interactive Nextstrain tree that visualizes the titers alongside all mutations.

A subclade K strain with mutations G142E and S193F was more potently neutralized by most sera than the parental subclade K strain (Figure 2). This increased neutralization by most sera is likely because S193F reverts this antigenic region B site to its amino-acid identity prior to ~2020; consistent with this hypothesis, the K:G142E,S193F strain is neutralized similarly to other subclade K strains by sera from young children (less than five years of age) who would not have been exposed to F193-carrying viruses or vaccines (see the interactive version of Figure 2 at https://jbloomlab.github.io/flu-seqneut-2026/summary.html that allows subsetting of sera by subject age using the sliders below the plot).

### H1N1 subclade D.3.1.1 has reduced neutralization, with Sa mutations strongly affecting a subset of sera

**Figure 4.**
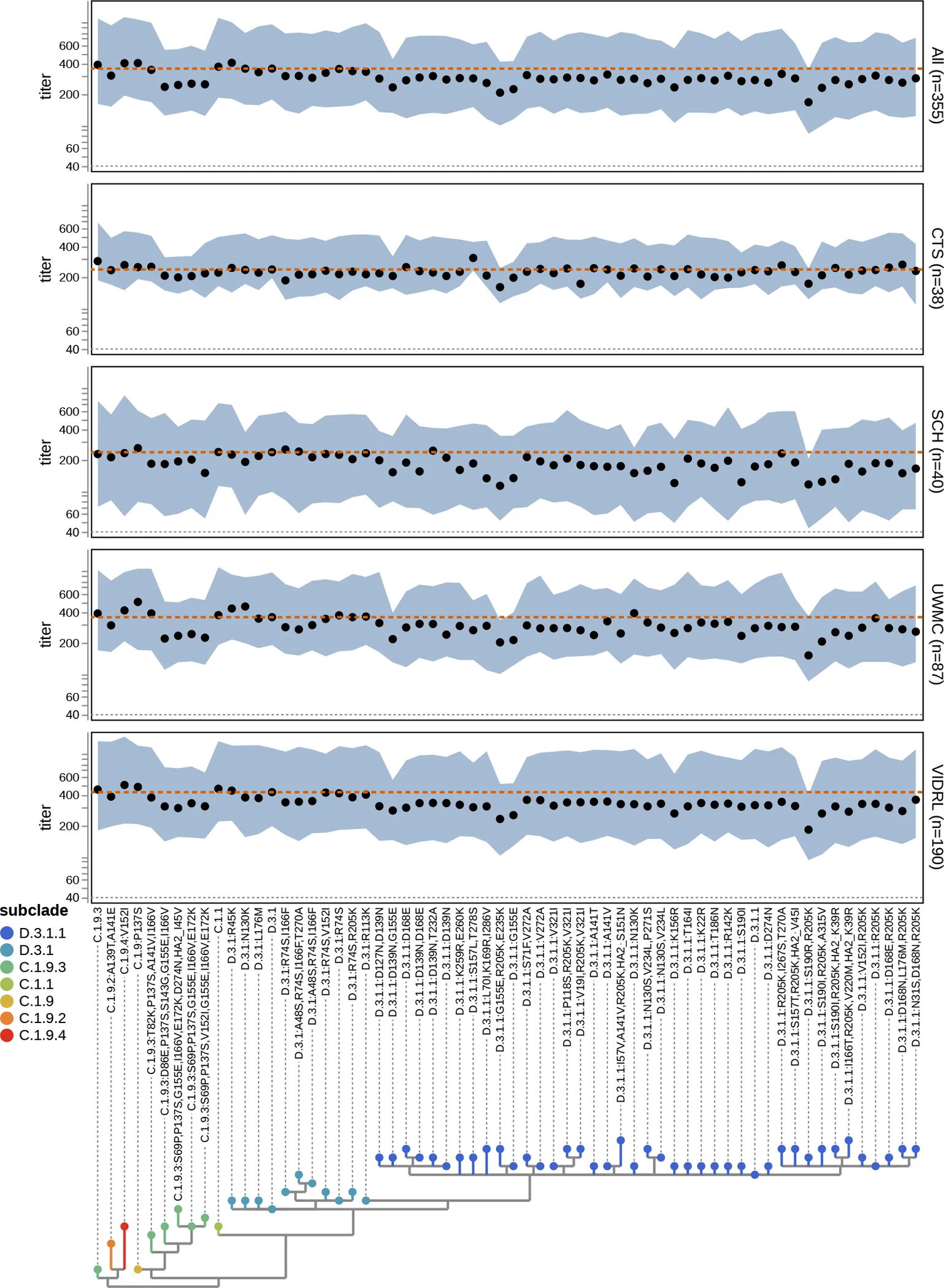
Neutralization titers of all tested sera against recent H1N1 HAs. Points indicate median titer and shaded regions indicate interquartile range. The dashed orange line indicates the titer against subclade D.3.1 (the 2026 and 2026–2027 cell-based vaccine strain), and the dotted gray line indicates the lower limit of detection (a titer of 40). Labels to the right of each plot indicate the sera set and number of sera in that set. X-axis labels indicate the HA protein haplotype (subclade and any derived mutations), and the phylogenetic tree shows the relationships among the protein sequences and is colored by subclade.

The neutralization titers against recent H1N1 strains are shown in Figure 4 and reported in Supplementary File 3; see also the summary website for interactive plots.

The 2026 Southern Hemisphere and 2026–2027 Northern Hemisphere H1N1 vaccine strain is a subclade D.3.1 strain, but nearly all recent human H1N1 sequences are from the D.3.1-descended subclade D.3.1.1. Most D.3.1.1 strains are neutralized more poorly by recent human sera than D.3.1 (below the dashed orange line in Figure 4). A handful of highly mutated strains descended from subclade C.1.9.3 are also poorly neutralized (Figure 4), although C.1.9.3 strains have not recently been observed in actual sequencing of human H1N1 influenza.

The lowest titers are to new D.3.1.1 strains with additional HA mutations (Figure 4). The strain with the lowest overall titers is D.3.1.1:S190R,R205K. Strains with G155E and to a lesser extent S157L (both in antigenic region Sa) also had clearly reduced titers. See https://nextstrain.org/community/jbloomlab/flu-seqneut-2026@main/H1N1 for an interactive Nextstrain tree that visualizes the titers alongside all mutations.

**Figure 5.**
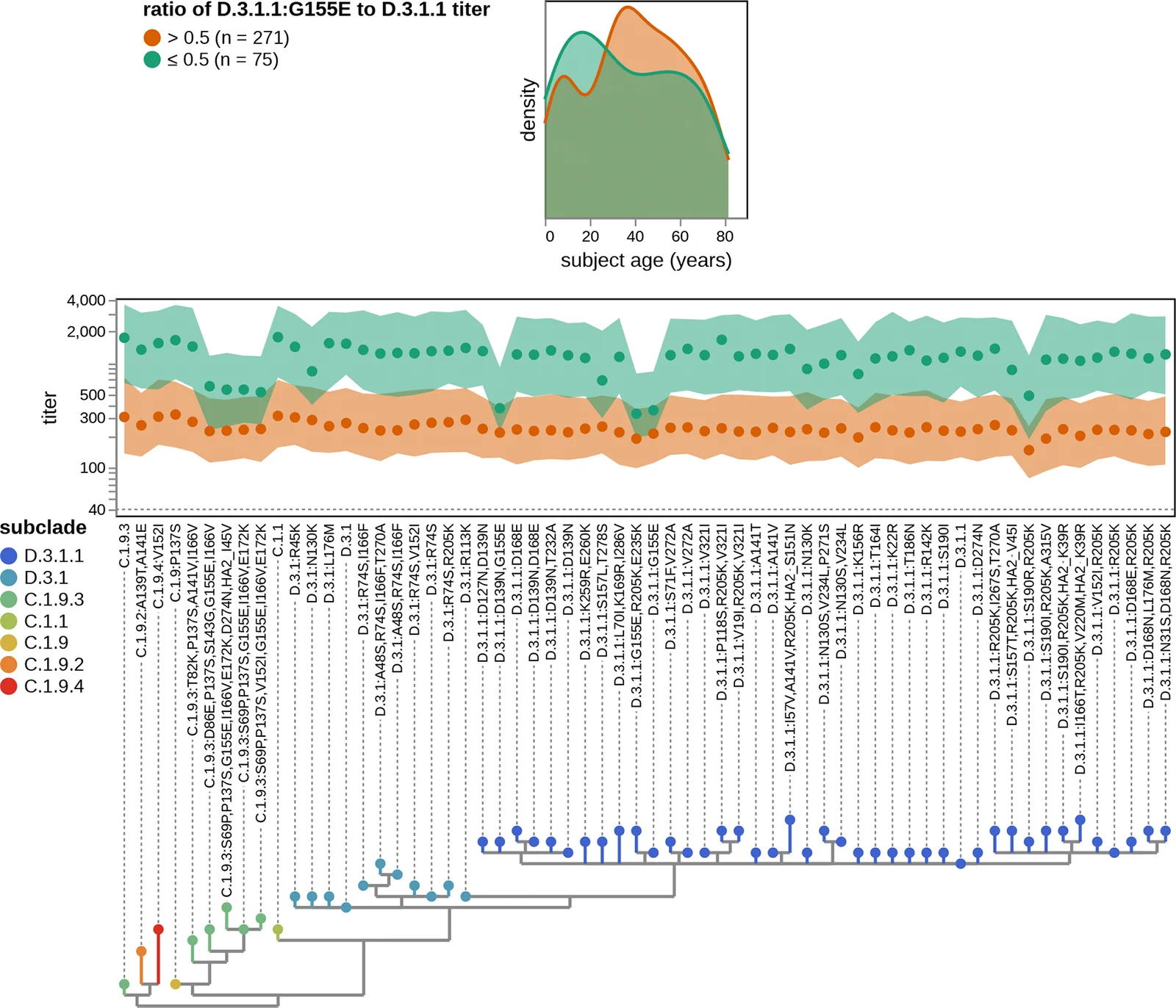
G155E in H1N1 HA causes a large decrease in neutralization for a subset of individuals who otherwise have high titers and tend to be teenagers or young adults. All sera stratified by whether the G155E mutation in D.3.1.1 reduces neutralization titers by more than two-fold relative to D.3.1.1 without any additional mutations. The top density plot shows the age distributions for both groups, and the lower plot shows the median (points) and interquartile range (shaded region) neutralization titers against recent H1N1 strains. In both plots, green indicates the 75 sera for which G155E causes a more than two-fold titer drop, and orange indicates the 271 sera for which G155E does not cause such a large titer drop. The dotted gray line in the titer plot indicates the lower limit of detection (a titer of 40). The handful of sera for which the titer against either D.3.1.1 or D.3.1.1:G155E was at the lower limit of detection are not shown in this plot to avoid confounding effects due to truncated measurements.

**Figure 6.**
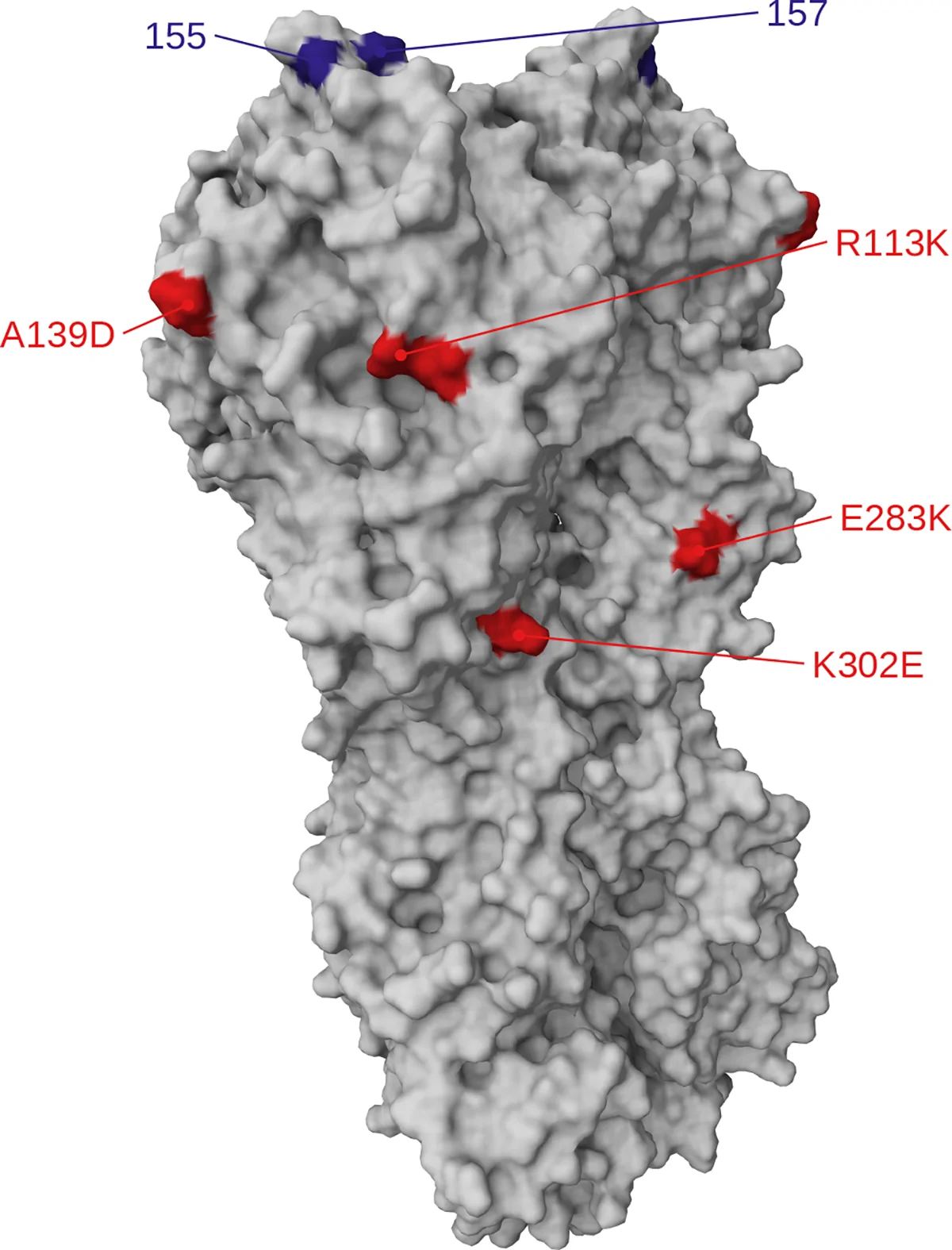
Structure of H1 HA indicating sites 155 and 157 and mutations that separate the 2026 and 2026–2027 cell-based vaccine (D.3.1) and subclade D.3.1.1. Structure of H1 trimer (PDB 9gsp) with red indicating mutations that separate D.3.1 from D.3.1.1, and blue indicating sites 155 and 157 where new mutations in D.3.1.1 further reduce neutralization. Site 139 is in antigenic region Ca2; sites 155 and 157 are in antigenic region Sa.

While strains with G155E are neutralized less well across all sera (Figure 4), the pattern is particularly striking if the sliders are used to subset on sera from individuals ages 15 to 25 years in the interactive plots at https://jbloomlab.github.io/flu-seqneut-2026/summary.html. The same trend can be seen in Figure 5, which stratifies sera based on whether or not D.3.1.1:G155E is neutralized at least two-fold worse than D.3.1.1. Sera that are strongly affected by G155E tend to be from teenagers and young adults and have higher overall H1N1 titers, but dramatically decreased titers to G155E-containing strains (Figure 5). A similar trend occurs to a lesser extent for S157L. Sites 155 and 157 are adjacent in antigenic region Sa at a structural location distinct from the other mutations that separate subclade D.3.1.1 from its parent subclade D.3.1 (Figure 6). Interestingly, G155E was described shortly after the 2009 H1N1 pandemic as a mutation that greatly reduced neutralization by sera from ferrets immunized with an early 2009 H1N1 pandemic strain but had little impact on sera from humans imprinted with pre-2009 H1N1 (Li et al. 2013; Chen et al. 2010, note G155E is G158E in H3 numbering). The age group in which G155E has the largest effect in our data is humans who were likely first exposed to H1N1 during or shortly after the 2009 pandemic, whereas the older individuals in which it has less effect would have been imprinted with the older H1N1 strains that preceded the 2009 pandemic. Notably, G155E has recently spread at high frequency in multiple D.3.1.1 H1N1 sub-lineages after previously remaining rare among human H1N1 influenza since 2009.

### The 2026 Southern Hemisphere vaccine boosts titers to all current H3N2 and H1N1 strains, with modest strain-to-strain variation

The Australia-based VIDRL sera were from four groups sampled before and after administration of the 2026 Southern Hemisphere influenza vaccine: children who received the egg-based Fluzone vaccine, adults who received the egg-based Fluzone vaccine, adults who received the cell-based Flucelvax vaccine, and elderly individuals who received the adjuvanted egg-based Fluad vaccine (Table 1 and Supplementary File 2). In addition to the differences in age and vaccine formulations, these groups differ in two other factors that can affect vaccine response: the timing of the post-vaccination serum collection (Lane et al. 2025) and extent to which they had been vaccinated in prior years (Cowling et al. 2024) (Table 1). Therefore, we show the adult Fluzone cohort stratified by prior-year vaccination status. Even so, we focus on trends in the strain-specific response within groups rather than on differences in the absolute titer increase after vaccination across groups, since the latter property could be affected by multiple factors.

**Figure 7.**
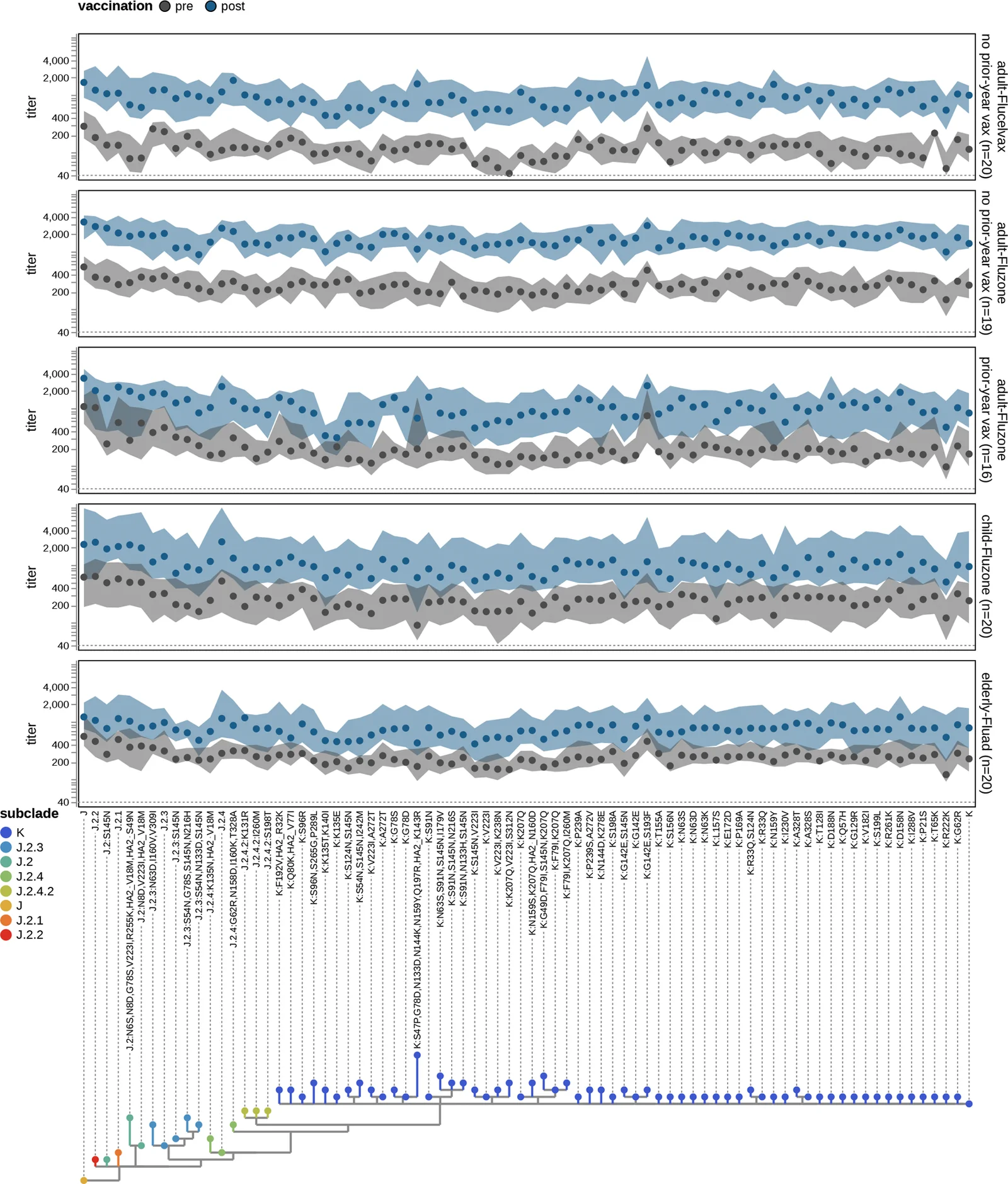
Neutralization titers pre- and post-vaccination against recent H3N2 HAs. Each plot shows the titers for a different cohort from the VIDRL (Australia) sera set before and after vaccination with the 2026 Southern Hemisphere vaccine (J.2.4 for the cell-based Flucelvax vaccine, and J.2.4:F195Y for the egg-based Fluzone and Fluad vaccines). The adult Fluzone cohort is further stratified by prior-year vaccination status; all adult Flucelvax recipients were not vaccinated in the prior year. Points indicate the median and shaded regions indicate the interquartile range, with gray indicating pre-vaccination titers and blue indicating post-vaccination titers. The dotted gray line indicates the lower limit of detection (a titer of 40). Post-vaccination sera were collected a median of three weeks after vaccination for the adult and elderly cohorts and five weeks after vaccination for the child sera; see Table 1.

**Figure 8.**
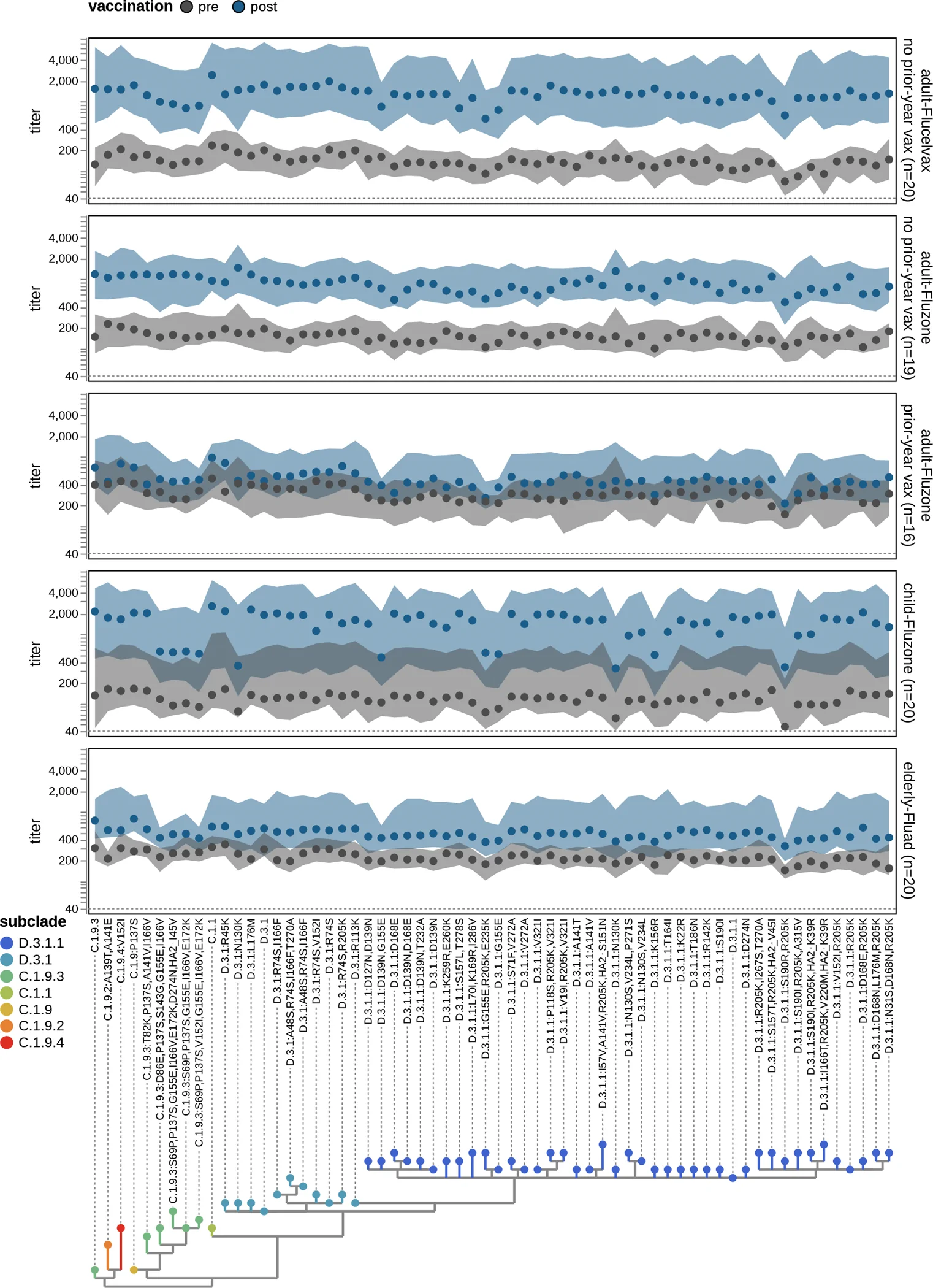
Neutralization titers pre- and post-vaccination against recent H1N1 HAs. Each plot shows the titers for a different cohort from the VIDRL (Australia) sera set before and after vaccination with the 2026 Southern Hemisphere vaccine (D.3.1 for the cell-based Flucelvax vaccine, and D.3.1:Q223R for the egg-based Fluzone and Fluad vaccines). The adult Fluzone cohort is further stratified by prior-year vaccination status; all adult Flucelvax recipients were not vaccinated in the prior year. Points indicate the median and shaded regions indicate the interquartile range, with gray indicating pre-vaccination titers and blue indicating post-vaccination titers. The dotted gray line indicates the lower limit of detection (a titer of 40). Post-vaccination sera were collected a median of three weeks after vaccination for the adult and elderly cohorts and five weeks after vaccination for the child sera; see Table 1.

Vaccination increased titers to all recent strains for both H3N2 and H1N1, so the post-vaccination strain-specific titers largely conform to the overall trends described in the preceding two sections (see Figure 7 and Figure 8 for raw titers, and the summary website for interactive plots that also show the fold change in titer after vaccination). The H3N2 strain in the 2026 Southern Hemisphere vaccine is J.2.4 for the cell-based vaccine and J.2.4:F195Y for the egg-based vaccine, and the H1N1 strain is D.3.1 for the cell-based vaccine and D.3.1:Q223R for the egg-based vaccine. However, vaccination did not induce a notably larger increase in titers against the matched J.2.4 and D.3.1 strains compared to more antigenically advanced subclade K and D.3.1.1 strains. Instead, the strain-specific post-vaccination titers mostly mirrored the pre-vaccination titers but generally shifted higher (Figure 7 and Figure 8). For H3N2 there were no strains to which vaccination consistently induced a lower titer boost across groups: for instance, the adult Fluzone group with a prior-year vaccination had a low titer boost to K:K135E, but other groups (including adults who received Flucelvax, children who received Fluzone, and adults receiving Fluzone without a prior-year vaccination) had a similar titer increase to the K:K135E as to other strains. For H1N1 there was a discernible trend for vaccination to induce a slightly lower titer boost to some strains (particularly those containing G155E), but the magnitude of this trend was modest.

## Discussion

We have measured how a large set of human sera neutralize current human H3N2 and H1N1 influenza strains. Our results show that the two subclades that spread widely over the last year (subclade K for H3N2 and D.3.1.1 for H1N1) are now spawning descendants with reduced neutralization by human sera. These descendant strains have HA mutations that are associated with decreased neutralization, some of which have arisen independently in different combinations. For H3N2, mutations at sites in antigenic region D (e.g., 223 and 222) have arisen recurrently and reduce neutralization; mutations at sites in antigenic regions A and B are also present in strains with reduced neutralization (e.g., 145, 156, 157). For H1N1, strains with mutations at sites in antigenic region Sa (e.g., 155 and 157) have arisen recurrently and reduce neutralization; mutations at sites in antigenic regions Sb and Ca1 (e.g., 190 and 205) are also present in strains with reduced neutralization.

However, the diversity of new antigenic variant strains makes it challenging to use our data alone to determine which strains will dominate a year from now. For H1N1, our data show that D.3.1.1 is antigenically advanced over D.3.1, and virtually all human H1N1 influenza observed over the last few months is D.3.1.1 or a descendant strain. However, it is uncertain which more antigenically advanced variant of H1N1 will spread over the next year—G155E reduces neutralization of all H1N1 strains and has recently arisen recurrently, but it occurs in multiple genetic backgrounds and there are also other antigenic mutations that could outcompete it. Similarly, although our data identify multiple new antigenic variant strains of subclade K, it remains unclear which of these new variants will dominate over the next year. Ultimately answering this question will require integrating neutralization data with forecasting models that also draw on other data sources (Łuksza and Lässig 2014; Huddleston et al. 2020; Shi et al. 2025; Neher et al. 2016; Chen et al. 2026)—such as sequencing counts (Abousamra et al. 2024) and mutation recurrence (Turner et al. 2026; Bloom and Neher 2023)—to estimate which current strains are most likely to spread in the human population.

Another finding is that despite clear antigenic variation among recent strains, the current 2026 Southern Hemisphere vaccine induced mostly similar increases in neutralization titer to all strains—such that the strain-to-strain variation in post-vaccination titers largely mirrored the variation in pre-vaccination titers. This raises the question of whether a vaccine update is merited even if it is possible to forecast which new strain will spread in the next year. However, we only measured neutralization by serum collected three to five weeks after vaccination when the boosted serum antibodies in individuals with a prior influenza exposure derive largely from a recall response of pre-existing memory B cells (Wrammert et al. 2008; Andrews et al. 2015; Ellebedy et al. 2016)—but influenza vaccines can also recruit naive strain-specific B-cells into germinal centers or lead to affinity maturation (Ellebedy et al. 2020; Turner et al. 2020; Matz et al. 2026; McIntire et al. 2024; Hoehn et al. 2021), a process that might not lead to an immediate change in serum titers. Studies of COVID-19 vaccination found that while a single updated booster vaccination of previously exposed individuals increased titers to both older and newer strains, over multiple years updated boosters partially overcame immune imprinting to preferentially increase titers to new viral variants (Yisimayi et al. 2024; Mellis et al. 2026; Tortorici et al. 2026; Alsoussi et al. 2023). So an updated vaccine can in principle lead to changes in the B-cell compartment that do not strongly manifest at the serological level until later exposures (Schiepers et al. 2023; Yisimayi et al. 2024), although it is unclear if this is actually the case for current influenza vaccines (Davis et al. 2020; Spangler et al. 2025).

Overall, our results underscore the complex heterogeneity of the human neutralizing antibody landscape (Cobey and Hensley 2017; Lee et al. 2019; Kikawa et al. 2026b). For instance, G155E in H1N1 appears to only modestly decrease neutralization if one simply examines the median across all sera—but in fact this mutation greatly decreases neutralization for some sera but only mildly impacts other sera. Here we have summarized a rich dataset full of such person-to-person heterogeneity as best as possible on the near real-time schedule required to make it useful for pending vaccine update decisions. But an important area for future work is analyzing these data as well as similar data from earlier timeframes (Kikawa et al. 2025; Kikawa et al. 2026a) to better understand how heterogeneous human neutralization data can inform forecasts of viral antigenic evolution.

## Methods

### Data and code availability

All data and computer code are at https://github.com/jbloomlab/flu-seqneut-2026. See https://jbloomlab.github.io/flu-seqneut-2026/summary.html for a summary of the results with interactive plots and links to the underlying data, and https://jbloomlab.github.io/flu-seqneut-2026 for more extensive documentation of the neutralization data at a per-plate and per-serum level. The final QC-ed titer data reported in this manuscript are in the CSV files at https://github.com/jbloomlab/flu-seqneut-2026/tree/main/results/final_titer_data and also in Supplementary File 1, Supplementary File 2, and Supplementary File 3.

### Biosafety

All experiments used viruses with HA protein ectodomains identical to recently circulating human seasonal H3N2 and H1N1 strains available in public databases (GenBank or GISAID) or recent cell- or egg-based vaccine strains. None of the HA ectodomains contained any additional protein mutations relative to recent human strains or vaccine strains. The rest of the HA gene segment and the seven non-HA genes were derived from the lab-adapted A/WSN/1933 (H1N1) strain. Since both recent human seasonal strains and the lab-adapted A/WSN/1933 strain are classified as appropriate for study at biosafety-level 2 by the BMBL handbook (edition 6), this work was approved by the Fred Hutch institutional biosafety committee at biosafety-level 2.

### HA numbering and definition of antigenic regions

Throughout this manuscript we use H3 numbering for the H3N2 HAs and H1 numbering for the H1N1 HAs (these numbering schemes start with 1 for the first residue of the ectodomain of that subtype, and number HA1 and HA2 separately). The antigenic region definitions are taken from (Stray and Pittman 2012) for H3N2 (see https://jbloomlab.github.io/flu-seqneut-2026/H3_prot_struct_viz.html#view=antigenic-regions for these regions projected on the HA structure and their constituent sites defined), and from (Wilson et al. 2015) for H1N1 (see https://jbloomlab.github.io/flu-seqneut-2026/H1_prot_struct_viz.html#view=antigenic-regions for these regions projected on the HA structure and their constituent sites defined).

### Human sera and plasma

Details on the sera, including the collection date and age of the individual from whom it was obtained, are provided in Table 1 and Supplementary File 2.

The VIDRL adult and elderly sera were collected by two sites with funding provided by Seqirus Limited for another study titled “A Sero-Epidemiological Study for the Collection of Pre- and Post-Vaccination Blood Samples from Volunteers Receiving a Southern Hemisphere Formulation of Inactivated Influenza Vaccine Recommended for the Season”; the de-identified sera reagents were then shared for the current study. Human research ethics approvals for this study were from the Adelaide University Women’s and Children’s Health Network Human Research Ethics Committee (application number 2020/HRE01220) and the University of Sunshine Coast Clinical Trials Bellberry Human Research Ethics Committee (application number 2025–12-2108).

The VIDRL child sera were collected from a single site with funding provided by AstraZeneca for another study titled “SNIF-FLES: Southern Hemisphere Nasal Influenza Flu Vaccine Experience Study”; the de-identified sera reagents were then shared for the current study. Human research ethics approval for this study was from Murdoch Children’s Research Institute, Melbourne (application number HREC/120054/RCHM-2026).

The SCH sera were obtained from children not known to be immunocompromised receiving medical care at Seattle Children’s Hospital, mostly for reasons unrelated to respiratory viral infection. The sera were obtained with a signed waiver of consent and the approval of the Seattle Children’s Hospital Institutional Review Board.

The CTS plasma samples were collected from volunteer adult blood donors across regions of the United States through a biobank maintained at Creative Testing Solutions by a collaboration between Vitalant Research Institute and the American Red Cross.

The deidentified remnant sera from UWMC were obtained from adults testing positive for HBsAb (to identify individuals with intact immunity) with approval from the University of Washington Institutional Review Board with a consent waiver.

All plasma and sera samples were processed identically before use in sequencing-based neutralization assays. Plasma samples were spun at 2000g for 10 minutes and cleared supernatant was transferred to new tubes. All sera and plasma were then treated with receptor-destroying enzyme II and heat-inactivated following a previously described protocol (Lee et al. 2019) to remove sialylated compounds that can neutralize virus by binding to the HA receptor-binding pocket, and to inactivate complement proteins. Briefly, lyophilized receptor-destroying enzyme II (Seiken) was resuspended in sterile phosphate-buffered saline (PBS) per manufacturer instructions and then passed through a 0.22 um filter before being aliquoted and frozen at −20C. These aliquots were thawed immediately prior to use, never undergoing more than a single freeze-thaw cycle. 25 uL of clarified plasma or sera was then incubated with 75 uL of receptor-destroying enzyme in 96-well PCR plates (Thermo Scientific) at 37C for 2.5 hours and then 55C for 30 minutes. All sera and plasma were used immediately following receptor-destroying enzyme II-treatment or stored at 4C until use. The titers reported here account for this initial four-fold dilution of the sera or plasma during this treatment.

### Choice of HAs for the sequencing-based neutralization assays

Our process for choosing HAs for the sequencing-based neutralization assay library aimed to select HAs that would be representative of circulating diversity in H1N1 and H3N2 HAs during the 2027 Southern Hemisphere influenza season. Due to the experimental time requirements for generating this library and running the neutralizations assays prior to the vaccine-selection meeting in September 2026, we made these selections largely based on data available in late May 2026. We first identified all HA protein haplotypes present in sequenced human influenza using the latest Nextstrain pdmH1N1 and H3N2 6-month builds available in May 2026 (Hadfield et al. 2018). We then created a ranked list of all haplotypes for each subtype based on a weighted combination of the following features: the total number of HA1 mutations, HA mutations in previously defined antigenically important sites or sites in/near the receptor binding site (Koel et al. 2013; Wolf et al. 2006; Caton et al. 1982), HA mutations arising at a rate greater than expected given the underlying mutation rate (Bloom and Neher 2023; Turner et al. 2026), the relative growth advantage of the haplotype compared with other concurrently circulating strains as defined by Nextstrain’s multinomial logistic regression forecast model (Abousamra et al. 2024), and the degree of geographic spread of the haplotype. The code for generating these lists of HA haplotypes is available at https://github.com/nextstrain/seasonal-flu/blob/fc2dd65/scripts/summarize_haplotypes_for_library_design.py. We used these ranked lists along with expert selection and curation to choose an initial set of 77 H3N2 haplotypes and 63 H1N1 haplotypes. Documentation and logic describing the design of the barcoded HA constructs is at https://github.com/jbloomlab/flu-seqneut-2026/tree/main/non-pipeline_analyses/library_design/full_design.

Additionally, this library was designed to cover the recent component strains for both the H1N1 and H3N2 seasonal vaccine strains. For H3N2, we included cell- and egg-based vaccine strains from the 2024 Southern Hemisphere vaccine to the 2026–2027 Northern Hemisphere vaccine. For H1N1, we attempted to include cell- and egg-based vaccine strains from the 2020–2021 Northern Hemisphere vaccine to the 2026–2027 Northern Hemisphere vaccine. However, not all H1N1 egg-based vaccine strains within this time frame were included in the library design as several do not grow well in the mammalian cell culture system used for our experiments; specifically, the following H1N1 vaccine strains were not included: A/Guangdong-Maonan/SWL1536/2019 (egg), A/Victoria/2570/2019 (egg), A/Sydney/5/2021 (egg and cell), and A/Victoria/4897/2022 (egg).

Overall, this design process resulted in a set of 154 HAs (most of which are associated with multiple distinct barcodes) that were included in the original designed library (see https://github.com/jbloomlab/flu-seqneut-2026/blob/main/data/viral_libraries/flu-seqneut-2026-barcode-to-strain-designed.csv). The set of 148 HAs that were actually included in viruses that could be generated at sufficient titers for use in the sequencing-based neutralization assays is at https://github.com/jbloomlab/flu-seqneut-2026/blob/main/data/viral_libraries/flu-seqneut-2026-barcode-to-strain-actual.csv and in Supplementary File 1.

### Production and pooling of viruses for sequencing-based neutralization assays

The sequencing-based neutralization assays measure titers via barcode sequencing, requiring HA genes to be linked to nucleotide barcodes. As in prior work (Bacsik et al. 2023; Welsh et al. 2024; Kikawa et al. 2026b; Loes et al. 2024), we linked HA genes to 16-nucleotide barcodes without disrupting genome packaging by duplicating the region at the end of the coding sequence containing the packaging signals. Randomly generated 16-nucleotide barcodes were cross-checked to avoid barcode collision (with other barcoded HA constructs from prior libraries) and barcodes starting with ‘GG’ (which we have found sequence poorly). The barcoded HA genes were then cloned into a plasmid backbone by Twist Biosciences or Genscript. For both H3N2 and H1N1 HA plasmids we used either a derivative of the pHH21 unidirectional reverse genetics plasmid (Neumann et al. 1999) or the pHW2000 bidirectional reverse genetics plasmid (Hoffmann et al. 2000). See https://github.com/jbloomlab/flu-seqneut-2026/blob/main/non-pipeline_analyses/data/example_vRNA_eGFP.gb for a Genbank file with the full annotated HA vRNA containing GFP in place of HA (used as the template for cloning), and https://github.com/jbloomlab/flu-seqneut-2026/blob/main/non-pipeline_analyses/data/example_vRNA_barcoded_H1_HA.gb or https://github.com/jbloomlab/flu-seqneut-2026/blob/main/non-pipeline_analyses/data/example_vRNA_barcoded_H3_HA.gb for example barcode HA vRNAs with a H1 or H3 HA ectodomain. While the bulk of the library was newly created, some constructs were generated in previous studies (Kikawa et al. 2025; Kikawa et al. 2026a; Loes et al. 2026; Kikawa et al. 2026b).

The barcoded HA plasmids were then used to individually generate barcoded virions using reverse genetics, which were then pooled together for use in sequencing-based neutralization assays. All HA genes were barcoded in duplicate or triplicate, and these replicate barcoded plasmids were pooled together prior to use. As previously described, each barcoded HA construct was transfected with all other non-HA genes from the lab-adapted A/WSN/1933 strain (see Biosafety subsection above) onto a co-culture of 293T and MDCK-SIAT1-TMPRSS2 and rescued 72 hours post transfection. These rescued viruses were then individually passaged on MDCK-SIAT1-TMPRSS2 cells.

The virus library pools used in sequencing-based neutralization assays should contain each barcoded virus at roughly equal proportions. As described previously, we first made an equal-volume pool of each individually generated and passaged virus and then titrated across MDCK-SIAT1 cells to determine each virus’s relative transcriptional titer (see report here: https://jbloomlab.github.io/flu-seqneut-2026/20260715_equal_vol_pool_analyze_pool.html). Using these estimated transcriptional titers, we repooled and re-titrated the pool to confirm the pool was more equally balanced and to determine the virus dilutions where viral transcription increased proportionally with the amount of virus infected on cells (see report here: https://jbloomlab.github.io/flu-seqneut-2026/20260810_repool_wash_spikein_lysis_measure_repool.html). Based on this analysis, the viral pool was used at a 1:64 dilution, since this was the highest virus pool concentration that was clearly in the linear range of viral barcode transcription as a function of infectious viral dose, as determined by dilution series analysis at https://jbloomlab.github.io/flu-seqneut-2026/20260810_repool_wash_spikein_lysis_measure_repool.html (the sequencing based readout requires transcription to scale linearly with viral infectivity).

The presence of virions containing different barcoded HAs in their genome than expressed on particle surface as a result of cross-contamination would be a major confounder for titers measured in the sequencing-based neutralization assay. To test for this type of cross-contamination, we individually infected each passaged virus on 50,000 MDCK-SIAT1 cells in a single well of a 96-well plate. At 16 hours post infection, we extracted viral RNA and sequenced per normal protocol for sequencing-based neutralization assays (see below). The results of that report are here (https://jbloomlab.github.io/flu-seqneut-2026/20260730_single_well_analyze_single_well_infections.html). This analysis confirmed none of the viral strains used in our experiments had appreciable contamination.

### Sequencing-based neutralization assays

The neutralization assays were performed as described previously (Kikawa et al. 2025; Kikawa et al. 2026a). The detailed protocol for these assays is available at https://doi.org/10.17504/protocols.io.kqdg3xdmpg25/v2; all details including the cell line, cell numbers, timepoints, etc are described in that protocol. As described in that protocol, infectivity is quantified by sequencing the viral barcodes in infected cells, and normalizing the barcode counts to an RNA standard consisting of known barcodes that is added at the cell lysis stage. Normalization to this RNA standard allows conversion of barcode sequencing counts to fraction viral infectivity retained at each serum concentration (Loes et al. 2024).

The initial serum dilution was 1:40 (accounting for the initial 1:4 dilution from the receptor-destroying enzyme treatment described above), making that the lower limit of detection for the assays. Matching plate formatting from prior iterations of this experiment (Kikawa et al. 2025; Kikawa et al. 2026a), sera were then diluted 2.3-fold across each 8-well column of a 96-well plate, making the upper limit of detection 1:13,620. The twelfth column of every 96-well plate was used for no-serum control wells.

### Analysis of sequencing-based neutralization assay data

We analyzed the sequencing-based neutralization assay data using *seqneut-pipeline* (https://github.com/jbloomlab/seqneut-pipeline), version 9.2.2. This pipeline uses *neutcurve* (https://github.com/jbloomlab/neutcurve) to fit neutralization curves to the fraction infectivity calculated from the barcode sequencing. It also applies quality control filters (configured at https://github.com/jbloomlab/flu-seqneut-2026/blob/main/config.yml) to remove low-quality neutralization curves. This filtering is why the final titer data set has 52,268 titers for the 148 viral strains measured against 355 sera rather than 148 × 355 = 52,540 titers: 272 curves were dropped during quality control. See https://github.com/jbloomlab/flu-seqneut-2026 for all computer code implementing the analysis, and https://jbloomlab.github.io/flu-seqneut-2026 that shows all measured curves for each plate and sera along with the full quality-control report.

All HA-expressing strains were barcoded in duplicate or triplicate (as described above), but in the analysis for this study we collapsed all barcode counts for the same HA by summing the counts—this is a different analysis approach than our prior studies (Kikawa et al. 2025; Kikawa et al. 2026a; Kikawa et al. 2026b; Loes et al. 2024), where we analyzed each barcode separately and then took the median of the measured titer for each barcode for the same strain. The potential benefit of analyzing each barcode for a strain separately is that the per-barcode replicate measurements provide multiple technical replicates for each HA neutralization curve. However, a primary source of experimental noise in the assay is that only a finite number of infectious particles for each variant are added to each well, and this number is two- to three-fold lower if variants are analyzed at the barcode rather than the strain level (since there are two to three barcodes per strain). Empirically we assessed that any benefits from having multiple barcode replicates for each strain were outweighed by the increased noise due to fewer infectious particles per variant per well when the data are analyzed on a per-barcode rather than per-strain level. Therefore, we added a collapse_strain_barcodes flag to the analysis pipeline and analyzed each strain as the sum of its barcode counts. See *seqneut-pipeline* documentation on the collapse_strain_barcodes flag for more details (https://github.com/jbloomlab/seqneut-pipeline/#collapse_strain_barcodes); note the flag’s value can be reversed in the main analysis pipeline to re-analyze the data on the barcode level, although we assess for this study that collapsing barcodes gives better-quality data.

For the figures in this paper, we show titers to all recent human strains and cell-based vaccine strains that were used as far back as the 2024 Southern Hemisphere influenza season. The figures in this paper do not show the titers to the egg-based vaccine strains or older cell-based vaccine strains. The reason is that we tend to measure higher neutralization titers of egg-based vaccine strains (Kikawa et al. 2026b) and older cell-based vaccine strains, so including them in the figures can “blow out” the scale and obscure the more relevant differences among recent strains. However, the titers to older vaccine strains and egg-based vaccine strains are plotted at https://jbloomlab.github.io/flu-seqneut-2026/summary.html#titers-to-older-and-egg-based-vaccine-strains and are provided in numerical form in the supplementary files and CSVs on the GitHub repository.

### Phylogenetic trees and subclade nomenclature

The phylogenetic trees available at https://nextstrain.org/community/jbloomlab/flu-seqneut-2026@main/H3N2 and https://nextstrain.org/community/jbloomlab/flu-seqneut-2026@main/H1N1 and shown along the x-axis of some figures in this paper are built on the HA protein sequences of the strains included in the library, and have branch lengths that represent the number of amino-acid mutations. See https://github.com/jbloomlab/nextstrain-prot-titers-tree (which is included as a git submodule for the main project GitHub repository) for the code used to build these trees.

On the trees and in the figures, strains are labeled by their subclade from the nomenclature described in (Neher et al. 2026) plus any additional HA amino-acid mutations. Any mutations falling in the HA2 region are annotated as such, whereas mutations in the HA1 region have no additional annotations. For instance, K:V223I means the strain has the subclade K HA amino-acid sequence plus the V223I mutation in HA1, whereas K:Q80K,HA2_V77I means the strain has subclade K amino-acid sequence with Q80K in HA1 and V77I in HA2.

## Supplementary Material

Supplement 1 Supplementary File 1. Details about viral HAs used in this study.
This CSV is also available at https://github.com/jbloomlab/flu-seqneut-2026/blob/main/results/final_titer_data/human_viruses.csv

Supplement 2 Supplementary File 2. Details about sera used in this study.
This CSV is also available at https://github.com/jbloomlab/flu-seqneut-2026/blob/main/results/final_titer_data/human_sera.csv

Supplement 3 Supplementary File 3. Neutralization titers measured in this study.
This CSV is also available at https://github.com/jbloomlab/flu-seqneut-2026/blob/main/results/final_titer_data/human_titers.csv

## Figures and Tables

**Table 1.** Human sera used in this study. Vaccination status in prior years is not known for the SCH, UWMC, and CTS sera.

Cohort	Description	Individuals	Sera	Collection month in 2026, median (range)	Age in years, median (range)	Days postvaccination, median (range)	Vaccinated in prior year

SCH	residual sera, Seattle Children’s Hospital, USA	40	40	Jun (May–Jun)	7.5 (0.5–13)		
UWMC	HBsAb+ residual sera, University of Washington Medical Center, USA	87	87	Jun	43 (19–78)		
CTS	blood donors, Creative Testing Solutions, USA	38	38	May	53.5 (26–82)		
VIDRL_adult-cell_pre	VIDRL, Australia, adults, Flucelvax, pre-vaccination	20	20	May (Apr–Jun)	33.5 (19–59)		0%
VIDRL_adult-cell_post	VIDRL, Australia, adults, Flucelvax, post-vaccination	20	20	Jun (May–Jul)	33.5 (19–59)	21 (18–25)	0%
VIDRL_adult-egg_pre	VIDRL, Australia, adults, Fluzone, pre-vaccination	35	35	Apr (Apr–May)	44 (18–63)		45.7%
VIDRL_adult-egg_post	VIDRL, Australia, adults, Fluzone, post-vaccination	35	35	May (May–Jun)	44 (18–63)	21 (18–25)	45.7%
VIDRL_elderly-egg_pre	VIDRL, Australia, elderly, Fluad, pre-vaccination	20	20	Apr (Apr–May)	71 (65–78)		70%
VIDRL_elderly-egg_post	VIDRL, Australia, elderly, Fluad, post-vaccination	20	20	May	71 (65–78)	21 (19–25)	70%
VIDRL_child-egg_pre	VIDRL, Australia, children, Fluzone, pre-vaccination	20	20	May (Apr–Jul)	5 (2.1–9.8)		85%
VIDRL_child-egg_post	VIDRL, Australia, children, Fluzone, post-vaccination	20	20	Jun (May–Aug)	5 (2.1–9.8)	34 (28–42)	85%

**All sera**		**260**	**355**	**May (Apr–Aug)**	**38 (0.5–82)**		

## References

[R1] Abousamra E, Figgins M, and Bedford T (2024). “Fitness models provide accurate short-term forecasts of SARS-CoV-2 variant frequency”. PLOS Computational Biology 20.9, e1012443. DOI: 10.1371/journal.pcbi.1012443. PMC11410224 39241101

[R2] Alsoussi WB, Malladi SK, Zhou JQ, Liu Z, Ying B, Kim W, Schmitz AJ, Lei T, Horvath SC, Sturtz AJ, McIntire KM, Evavold B, Han F, Scheaffer SM, Fox IF, Mirza SF, Parra-Rodriguez L, Nachbagauer R, Nestorova B, Chalkias S, Farnsworth CW, Klebert MK, Pusic I, Strnad BS, Middleton WD, Teefey SA, Whelan SPJ, Diamond MS, Paris R, O’Halloran JA, Presti RM, Turner JS, and Ellebedy AH (2023). “SARS-CoV-2 Omicron boosting induces de novo B cell response in humans”. Nature 617.7961, pp. 592–598. DOI: 10.1038/s41586-023-06025-4. 37011668

[R3] Andrews SF, Huang Y, Kaur K, Popova LI, Ho IY, Pauli NT, Dunand CJH, Taylor WM, Lim S, Huang M, Qu X, Lee JH, Salgado-Ferrer M, Krammer F, Palese P, Wrammert J, Ahmed R, and Wilson PC (2015). “Immune history profoundly affects broadly protective B cell responses to influenza”. Science Translational Medicine 7.316. DOI: 10.1126/scitranslmed.aad0522. PMC4770855 26631631

[R4] Bacsik DJ, Dadonaite B, Butler A, Greaney AJ, Heaton NS, and Bloom JD (2023). “Influenza virus transcription and progeny production are poorly correlated in single cells”. eLife 12. DOI: 10.7554/elife.86852. PMC10484525 37675839

[R5] Bedford T, Riley S, Barr IG, Broor S, Chadha M, Cox NJ, Daniels RS, Gunasekaran CP, Hurt AC, Kelso A, Klimov A, Lewis NS, Li X, McCauley JW, Odagiri T, Potdar V, Rambaut A, Shu Y, Skepner E, Smith DJ, Suchard MA, Tashiro M, Wang D, Xu X, Lemey P, and Russell CA (2015). “Global circulation patterns of seasonal influenza viruses vary with antigenic drift”. Nature 523.7559, pp. 217–220. DOI: 10.1038/nature14460. PMC4499780 26053121

[R6] Bedford T, Suchard MA, Lemey P, Dudas G, Gregory V, Hay AJ, McCauley JW, Russell CA, Smith DJ, and Rambaut A (2014). “Integrating influenza antigenic dynamics with molecular evolution”. eLife 3. DOI: 10.7554/elife.01914. PMC3909918 24497547

[R7] Bloom JD and Neher RA (2023). “Fitness effects of mutations to SARS-CoV-2 proteins”. Virus Evolution 9.2. DOI: 10.1093/ve/vead055. PMC10506532 37727875

[R8] Boon ACM, de Mutsert G, Graus YMF, Fouchier RAM, Sintnicolaas K, Osterhaus ADME, and Rimmelzwaan GF (2002). “Sequence Variation in a Newly Identified HLA-B35-Restricted Epitope in the Influenza A Virus Nucleoprotein Associated with Escape from Cytotoxic T Lymphocytes”. Journal of Virology 76.5, pp. 2567–2572. DOI: 10.1128/jvi.76.5.2567-2572.2002. PMC153818 11836437

[R9] Caton AJ, Brownlee GG, Yewdell JW, and Gerhard W (1982). “The antigenic structure of the influenza virus A/PR/8/34 hemagglutinin (H1 subtype)”. Cell 31.2, pp. 417–427. DOI: 10.1016/0092-8674(82)90135-0. 6186384

[R10] Chen Y, Xu Y, Cheng Y, Qi X, Bai T, Yang J, Luo H, Du X, Zhu L, Yang L, Shi M, Wang D, Li Z, and Shu Y (2026). “A sequence-based proactive intelligence for influenza antigenic profiling improves vaccine strain selection”. DOI: 10.64898/2026.04.18.719333.

[R11] Chen Z, Wang W, Zhou H, Suguitan AL, Shambaugh C, Kim L, Zhao J, Kemble G, and Jin H (2010). “Generation of Live Attenuated Novel Influenza Virus A/California/7/09 (H1N1) Vaccines with High Yield in Embryonated Chicken Eggs”. Journal of Virology 84.1, pp. 44–51. DOI: 10.1128/jvi.02106-09. PMC2798434 19864389

[R12] Cheng SM, Leung NH, Shiu EY, Wong RW, Au NY, Huang X, Kwok NK, Chan CH, Li JK, Tsang LC, Kwan TC, Tsang TK, Poon LL, Cowling BJ, and Peiris M (2026). “Population immunity, post-vaccine and post-infection antibody responses to influenza A (H3N2) subclade J.2 and K (J.2.4.1) viruses, Hong Kong, 2025”. Eurosurveillance 31.10. DOI: 10.2807/1560-7917.es.2026.31.10.2600185. PMC13074472 41822984

[R13] Cobey S and Hensley SE (2017). “Immune history and influenza virus susceptibility”. Current Opinion in Virology 22, pp. 105–111. DOI: 10.1016/j.coviro.2016.12.004. PMC5467731 28088686

[R14] Cowling BJ, Wong SS, Santos JJS, Touyon L, Ort JT, Ye N, Kwok NKM, Ho F, Cheng SMS, Ip DKM, Peiris M, Webby RJ, Wilson PC, Valkenburg SA, Tsang JS, Leung NHL, Hensley SE, and Cobey S (2024). “Preliminary Findings From the Dynamics of the Immune Responses to Repeat Influenza Vaccination Exposures (DRIVE I) Study: A Randomized Controlled Trial”. Clinical Infectious Diseases 79.4, pp. 901–909. DOI: 10.1093/cid/ciae380. PMC11478574 39041887

[R15] Davis CW, Jackson KJL, McCausland MM, Darce J, Chang C, Linderman SL, Chennareddy C, Gerkin R, Brown SJ, Wrammert J, Mehta AK, Cheung WC, Boyd SD, Waller EK, and Ahmed R (2020). “Influenza vaccine–induced human bone marrow plasma cells decline within a year after vaccination”. Science 370.6513, pp. 237–241. DOI: 10.1126/science.aaz8432. PMC10155619 32792465

[R16] Dee K, Imrie RM, Mojsiejczuk L, Raveendran S, Chen H, Guio Archila G, Aljeri MS, Schultz V, Wang Z, Walsh SK, Zhang J, Hutchinson E, Willett BJ, Robertson DL, Hughes J, MacLean OA, Grove J, Thomson EC, Illingworth CJR, and Murcia PR (2026). “Subclade K influenza A (H3N2) viruses display partial immune escape with preserved cross-neutralisation in a UK population”. DOI: 10.64898/2026.01.27.26344933.

[R17] Ellebedy AH, Jackson KJL, Kissick HT, Nakaya HI, Davis CW, Roskin KM, McElroy AK, Oshansky CM, Elbein R, Thomas S, Lyon GM, Spiropoulou CF, Mehta AK, Thomas PG, Boyd SD, and Ahmed R (2016). “Defining antigen-specific plasmablast and memory B cell subsets in human blood after viral infection or vaccination”. Nature Immunology 17.10, pp. 1226–1234. DOI: 10.1038/ni.3533. PMC5054979 27525369

[R18] Ellebedy AH, Nachbagauer R, Jackson KJL, Dai YN, Han J, Alsoussi WB, Davis CW, Stadlbauer D, Rouphael N, Chromikova V, McCausland M, Chang CY, Cortese M, Bower M, Chennareddy C, Schmitz AJ, Zarnitsyna VI, Lai L, Rajabhathor A, Kazemian C, Antia R, Mulligan MJ, Ward AB, Fremont DH, Boyd SD, Pulendran B, Krammer F, and Ahmed R (2020). “Adjuvanted H5N1 influenza vaccine enhances both cross-reactive memory B cell and strain-specific naive B cell responses in humans”. Proceedings of the National Academy of Sciences 117.30, pp. 17957–17964. DOI: 10.1073/pnas.1906613117. PMC7395544 32661157

[R19] Hadfield J, Megill C, Bell SM, Huddleston J, Potter B, Callender C, Sagulenko P, Bedford T, and Neher RA (2018). “Nextstrain: real-time tracking of pathogen evolution”. Bioinformatics 34.23, pp. 4121–4123. DOI: 10.1093/bioinformatics/bty407. PMC6247931 29790939

[R20] Hobson D, Curry RL, Beare AS, and Ward-Gardner A (1972). “The role of serum haemagglutination-inhibiting antibody in protection against challenge infection with influenza A2 and B viruses”. Epidemiology and Infection 70.4, pp. 767–777. DOI: 10.1017/s0022172400022610. PMC2130285 4509641

[R21] Hoehn KB, Turner JS, Miller FI, Jiang R, Pybus OG, Ellebedy AH, and Kleinstein SH (2021). “Human B cell lineages associated with germinal centers following influenza vaccination are measurably evolving”. eLife 10. DOI: 10.7554/elife.70873. PMC8741214 34787567

[R22] Hoffmann E, Neumann G, Kawaoka Y, Hobom G, and Webster RG (2000). “A DNA transfection system for generation of influenza A virus from eight plasmids”. Proceedings of the National Academy of Sciences 97.11, pp. 6108–6113. DOI: 10.1073/pnas.100133697. PMC18566 10801978

[R23] Huddleston J, Barnes JR, Rowe T, Xu X, Kondor R, Wentworth DE, Whittaker L, Ermetal B, Daniels RS, McCauley JW, Fujisaki S, Nakamura K, Kishida N, Watanabe S, Hasegawa H, Barr I, Subbarao K, Barrat-Charlaix P, Neher RA, and Bedford T (2020). “Integrating genotypes and phenotypes improves long-term forecasts of seasonal influenza A/H3N2 evolution”. eLife 9. DOI: 10.7554/elife.60067. PMC7553778 32876050

[R24] Ikonen N, Haveri A, Lindh E, Liedes O, Vara S, Pakkanen SH, Kantele A, Nieminen T, Anttila VJ, Välimaa H, Melin M, Savolainen-Kopra C, and Nohynek H (2026). “Reduced neutralising antibody responses against emerging 2025/26 influenza A(H1N1)pdm09 subclade D.3.1 and A(H3N2) subclade K viruses among healthcare workers, Finland, August to October 2025”. Eurosurveillance 31.6. DOI: 10.2807/1560-7917.es.2026.31.6.2600094. PMC12905531 41684323

[R25] Kikawa C, Huddleston J, Loes AN, Turner SA, Lee J, Barr IG, Cowling BJ, Englund JA, Greninger AL, Harvey R, Hasegawa H, Ho F, Lacombe K, Leung NHL, Lewis NS, Peck H, Watanabe S, Smith DJ, Bedford T, and Bloom JD (2025). “Near real-time data on the human neutralizing antibody landscape to influenza virus to inform vaccine-strain selection in September 2025”. Virus Evolution 11.1. DOI: 10.1093/ve/veaf086. PMC12640540 41287634

[R26] Kikawa C, Huddleston J, Turner SA, Loes AN, Liu J, Gang S, Griffiths T, Drapeau EM, Cowling BJ, Ho F, Leung NHL, Englund JA, Lacombe K, Watanabe S, Hasegawa H, Busch M, Lanteri M, Stone M, Spencer B, Neher RA, Smith DJ, Bedford T, Hensley SE, and Bloom JD (2026a). “Near real-time data on the human neutralizing antibody landscape to influenza virus as of early 2026 to inform vaccine-strain selection”. Virus Evolution 12.1. DOI: 10.1093/ve/veag046. PMC13499528 42633301

[R27] Kikawa C, Loes AN, Huddleston J, Figgins MD, Steinberg P, Griffiths T, Drapeau EM, Peck H, Barr I, Englund JA, Hensley SE, Bedford T, and Bloom JD (2026b). “High-throughput neutralization measurements correlate strongly with evolutionary success of human influenza strains”. eLife 14. DOI: 10.7554/elife.106811.4. PMC12928701 41725518

[R28] Kim K, Vieira MC, Gouma S, Weirick ME, Hensley SE, and Cobey S (2024). “Measures of Population Immunity Can Predict the Dominant Clade of Influenza A (H3N2) in the 2017–2018 Season and Reveal Age-Associated Differences in Susceptibility and Antibody-Binding Specificity”. Influenza and Other Respiratory Viruses 18.11. DOI: 10.1111/irv.70033. PMC11538025 39501522

[R29] Kirsebom FC, Thompson C, Talts T, Kele B, Whitaker HJ, Andrews N, Abdul Aziz N, Rawlinson C, Green RE, Quinot C, Gardner N, Waller E, Allen A, Watson CH, McDonald SL, Zambon M, Pebody R, Ramsay M, Hoschler K, Singanayagam A, and Lopez Bernal J (2025). “Early influenza virus characterisation and vaccine effectiveness in England in autumn 2025, a period dominated by influenza A(H3N2) subclade K”. Eurosurveillance 30.46. DOI: 10.2807/1560-7917.es.2025.30.46.2500854. PMC12639273 41267661

[R30] Koel BF, Burke DF, Bestebroer TM, van der Vliet S, Zondag GCM, Vervaet G, Skepner E, Lewis NS, Spronken MIJ, Russell CA, Eropkin MY, Hurt AC, Barr IG, de Jong JC, Rimmelzwaan GF, Osterhaus ADME, Fouchier RAM, and Smith DJ (2013). “Substitutions Near the Receptor Binding Site Determine Major Antigenic Change During Influenza Virus Evolution”. Science 342.6161, pp. 976–979. DOI: 10.1126/science.1244730. 24264991

[R31] Krammer F (2019). “The human antibody response to influenza A virus infection and vaccination”. Nature Reviews Immunology 19.6, pp. 383–397. DOI: 10.1038/s41577-019-0143-6. 30837674

[R32] Lane A, Quach HQ, Ovsyannikova IG, Kennedy RB, Ross TM, and Einav T (2025). “High-resolution antibody dynamics following influenza vaccination reveal predominantly weak responses as well as infrequent but durable immunity across the 2014–2022 seasons”. Vaccine 64, p. 127677. DOI: 10.1016/j.vaccine.2025.127677. PMC12435915 40946064

[R33] Lee JM, Eguia R, Zost SJ, Choudhary S, Wilson PC, Bedford T, Stevens-Ayers T, Boeckh M, Hurt AC, Lakdawala SS, Hensley SE, and Bloom JD (2019). “Mapping person-to-person variation in viral mutations that escape polyclonal serum targeting influenza hemagglutinin”. eLife 8. DOI: 10.7554/elife.49324. PMC6711711 31452511

[R34] Li Y, Myers JL, Bostick DL, Sullivan CB, Madara J, Linderman SL, Liu Q, Carter DM, Wrammert J, Esposito S, (2013). “Immune history shapes specificity of pandemic H1N1 influenza antibody responses”. Journal of Experimental Medicine 210.8, pp. 1493–1500. DOI: 10.1084/jem.20130212. PMC3727314 23857983

[R35] Liu H, Shaw-Saliba K, Westerbeck J, Jacobs D, Fenstermacher K, Chao CY, Gong YN, Powell H, Ma Z, Mehoke T, Ernlund AW, Dziedzic A, Vyas S, Evans J, Sauer LM, Wu CC, Chen SH, Rothman RE, Thielen P, Chen KF, and Pekosz A (2024). “Effect of human H3N2 influenza virus reassortment on influenza incidence and severity during the 2017–18 influenza season in the USA: a retrospective observational genomic analysis”. The Lancet Microbe 5.8, p. 100852. DOI: 10.1016/s2666-5247(24)00067-3. PMC11338072 38734029

[R36] Liu J, Gang S, Kikawa C, Rodriguez AJ, Li SH, Ye N, Griffiths T, Drapeau EM, Atkinson RK, Loes AN, Collman RG, Ferguson JA, Han J, Ward AB, Bloom JD, and Hensley SE (2026a). “Mapping the specificity of H3N2 strain-specific and cross-reactive human neutralizing antibodies elicited by the 2025–2026 influenza vaccine”. DOI: 10.64898/2026.02.20.26346746.

[R37] Liu J, Li SH, Ye N, Griffiths T, Drapeau EM, Atkinson RK, Collman RG, and Hensley SE (2026b). “Antibodies Elicited by the 2025–2026 Influenza Vaccine”. NEJM Evidence 5.4. DOI: 10.1056/evidpha2600007. 41774915

[R38] Loes AN, Tarabi RAL, Huddleston J, Touyon L, Wong SS, Cheng SMS, Leung NHL, Hannon WW, Bedford T, Cobey S, Cowling BJ, and Bloom JD (2024). “High-throughput sequencing-based neutralization assay reveals how repeated vaccinations impact titers to recent human H1N1 influenza strains”. Journal of Virology 98.10. DOI: 10.1128/jvi.00689-24. PMC11494878 39315814

[R39] Loes AN, Tarabi RAL, Li SH, Atkinson RK, Huddleston J, Kikawa C, Griffiths T, Drapeau EM, Wong SS, Cheng SMS, Leung NHL, Cobey S, Cowling BJ, Bedford T, Hensley SE, and Bloom JD (2026). “Strain-specific differences in the response to egg-derived versus recombinant protein influenza vaccines”. Journal of Virology 100.6. DOI: 10.1128/jvi.00317-26. PMC13288920 42200639

[R40] Łuksza M and Lässig M (2014). “A predictive fitness model for influenza”. Nature 507.7490, pp. 57–61. DOI: 10.1038/nature13087. 24572367

[R41] Machkovech HM, Bedford T, Suchard MA, and Bloom JD (2015). “Positive Selection in CD8^+^T-Cell Epitopes of Influenza Virus Nucleoprotein Revealed by a Comparative Analysis of Human and Swine Viral Lineages”. Journal of Virology 89.22, pp. 11275–11283. DOI: 10.1128/jvi.01571-15. PMC4645657 26311880

[R42] Matz HC, Yu TG, Dixit K, Kikawa C, Zhou JQ, Pena Alzua G, Peyton L, Madsen A, Han F, Farrell AG, Hoelzl R, Schmitz AJ, Horvath SC, Keplinger HK, Strnad BS, Hoegger MJ, Middleton WD, Klebert MK, Lin NH, Nachbagauer R, Krammer F, Paris R, Bloom JD, Turner JS, Presti RM, Lee J, and Ellebedy AH (2026). “mRNA-based influenza vaccine expands the B cell response breadth in humans”. Nature Immunology 27.8, pp. 1666–1677. DOI: 10.1038/s41590-026-02569-5. PMC13414572 42297975

[R43] McIntire KM, Meng H, Lin TH, Kim W, Moore NE, Han J, McMahon M, Wang M, Malladi SK, Mohammed BM, Zhou JQ, Schmitz AJ, Hoehn KB, Carreño JM, Yellin T, Suessen T, Middleton WD, Teefey SA, Presti RM, Krammer F, Turner JS, Ward AB, Wilson IA, Kleinstein SH, and Ellebedy AH (2024). “Maturation of germinal center B cells after influenza virus vaccination in humans”. Journal of Experimental Medicine 221.8. DOI: 10.1084/jem.20240668. PMC11211068 38935072

[R44] Mellis IA, Wu M, Hong H, Bowen A, Daniel K, Gherasim C, Pierce VM, Yin MT, Gordon A, Guo Y, and Ho DD (2026). “LP.8.1-directed COVID-19 mRNA vaccines durably boost neutralizing antibodies and mitigate ancestral immune imprinting”. PLOS Pathogens 22.5, e1014218. DOI: 10.1371/journal.ppat.1014218. PMC13178986 42113876

[R45] Monto AS, Petrie JG, Cross RT, Johnson E, Liu M, Zhong W, Levine M, Katz JM, and Ohmit SE (2015). “Antibody to Influenza Virus Neuraminidase: An Independent Correlate of Protection”. Journal of Infectious Diseases 212.8, pp. 1191–1199. DOI: 10.1093/infdis/jiv195. 25858957

[R46] Neher RA, Bedford T, Daniels RS, Russell CA, and Shraiman BI (2016). “Prediction, dynamics, and visualization of antigenic phenotypes of seasonal influenza viruses”. Proceedings of the National Academy of Sciences 113.12. DOI: 10.1073/pnas.1525578113. PMC4812706 26951657

[R47] Neher RA, Huddleston J, Bedford T, Lewis NS, Harvey R, Galiano M, Byrne AMP, James S, Smith D, Łuksza M, Ruchnewitz D, Laessig M, Fujisaki S, Watanabe S, Hasegawa H, Hassell N, Wentworth DE, Kondor R, Deng YM, Dapat C, Subbarao K, and Barr I (2026). “Nomenclature for Tracking of Genetic Variation of Seasonal Influenza Viruses”. Influenza and Other Respiratory Viruses 20.2. DOI: 10.1111/irv.70230. PMC12904685 41688063

[R48] Neumann G, Watanabe T, Ito H, Watanabe S, Goto H, Gao P, Hughes M, Perez DR, Donis R, Hoffmann E, Hobom G, and Kawaoka Y (1999). “Generation of influenza A viruses entirely from cloned cDNAs”. Proceedings of the National Academy of Sciences 96.16, pp. 9345–9350. DOI: 10.1073/pnas.96.16.9345. PMC17785 10430945

[R49] Petrie JG, Parkhouse K, Ohmit SE, Malosh RE, Monto AS, and Hensley SE (2016). “Antibodies Against the Current Influenza A(H1N1) Vaccine Strain Do Not Protect Some Individuals From Infection With Contemporary Circulating Influenza A(H1N1) Virus Strains”. Journal of Infectious Diseases 214.12, pp. 1947–1951. DOI: 10.1093/infdis/jiw479. PMC5142093 27923954

[R50] Raghwani J, Thompson RN, and Koelle K (2017). “Selection on non-antigenic gene segments of seasonal influenza A virus and its impact on adaptive evolution”. Virus Evolution 3.2. DOI: 10.1093/ve/vex034. PMC5724400 29250432

[R51] Schiepers A, ’t Wout MFL van, Greaney AJ, Zang T, Muramatsu H, Lin PJC, Tam YK, Mesin L, Starr TN, Bieniasz PD, Pardi N, Bloom JD, and Victora GD (2023). “Molecular fate-mapping of serum antibody responses to repeat immunization”. Nature 615.7952, pp. 482–489. DOI: 10.1038/s41586-023-05715-3. PMC10023323 36646114

[R52] Separovic L, Sabaiduc S, Zhan Y, Kaweski SE, Olsha R, Hasso M, Mather RG, Carazo S, Lacroix C, Meunier I, Salhi LN, Dickinson JA, Zelyas N, Jassem AN, Dover K, Ranadheera C, Gao R, Bastien N, and Skowronski DM (2026). “Interim 2025/26 influenza vaccine effectiveness estimates with immuno-epidemiological considerations for A(H3N2) subclade K protection, Canada, January 2026”. Eurosurveillance 31.5. DOI: 10.2807/1560-7917.es.2026.31.5.2600068. PMC12881843 41645799

[R53] Shi W, Wohlwend J, Wu M, and Barzilay R (2025). “Influenza vaccine strain selection with an AI-based evolutionary and antigenicity model”. Nature Medicine 31.11, pp. 3862–3870. DOI: 10.1038/s41591-025-03917-y. PMC12618262 40877477

[R54] Shu Y and McCauley J (2017). “GISAID: Global initiative on sharing all influenza data – from vision to reality”. Eurosurveillance 22.13. DOI: 10.2807/1560-7917.es.2017.22.13.30494. PMC5388101 28382917

[R55] Smith DJ, Lapedes AS, de Jong JC, Bestebroer TM, Rimmelzwaan GF, Osterhaus ADME, and Fouchier RAM (2004). “Mapping the Antigenic and Genetic Evolution of Influenza Virus”. Science 305.5682, pp. 371–376. DOI: 10.1126/science.1097211. 15218094

[R56] Spangler A, Shimberg GD, Mantus GE, Malek R, Cominsky LY, Tsybovsky Y, Li N, Gillespie RA, Ravichandran M, Creanga A, Raab JE, Gajjala SR, Mendoza F, Houser KV, Dropulic L, McDermott AB, Kanekiyo M, and Andrews SF (2025). “Early influenza virus exposure shapes the B cell response to influenza vaccination in individuals 50 years later”. Immunity 58.3, 728–744.e9. DOI: 10.1016/j.immuni.2025.02.004. PMC11979964 40023164

[R57] Stray SJ and Pittman LB (2012). “Subtype- and antigenic site-specific differences in biophysical influences on evolution of influenza virus hemagglutinin”. Virology Journal 9.1. DOI: 10.1186/1743-422x-9-91. PMC3499391 22569196

[R58] Tortorici MA, Sprouse KR, Addetia A, Brown JT, Lee J, Stewart C, Merz B, Harteloo A, Elias-Warren A, Chu HY, and Veesler D (2026). “Repeated COVID-19 vaccine boosters elicit variant-specific memory B cells in humans”. Cell Reports 45.3, p. 117052. DOI: 10.1016/j.celrep.2026.117052. PMC13607844 41800618

[R59] Turner JS, Zhou JQ, Han J, Schmitz AJ, Rizk AA, Alsoussi WB, Lei T, Amor M, McIntire KM, Meade P, Strohmeier S, Brent RI, Richey ST, Haile A, Yang YR, Klebert MK, Suessen T, Teefey S, Presti RM, Krammer F, Kleinstein SH, Ward AB, and Ellebedy AH (2020). “Human germinal centres engage memory and naive B cells after influenza vaccination”. Nature 586.7827, pp. 127–132. DOI: 10.1038/s41586-020-2711-0. PMC7566073 32866963

[R60] Turner SA, Pattinson DJ, Fouchier RAM, and Smith DJ (2026). “Predicting the antigenic evolution of seasonal influenza viruses using phylogenetic convergence”. DOI: 10.64898/2026.04.10.717627.

[R61] Wang L, Nangle SJ, Waller-Pulido A, McMahan K, Pereira J, Chaudhari J, Barrett LO, Bhowmik R, Ferrugia K, Ramsamooj R, Sordillo EM, Simon V, van Bakel H, Mostafa H, Pekosz A, Collier ArY, and Barouch DH (2026). “Vaccine-Elicited Antibody Responses to Influenza H3N2 Subclade K”. DOI: 10.64898/2026.02.02.26345378. PMC12973212 41801760

[R62] Welsh FC, Eguia RT, Lee JM, Haddox HK, Galloway J, Van Vinh Chau N, Loes AN, Huddleston J, Yu TC, Quynh Le M, Nhat NT, Thi Le Thanh N, Greninger AL, Chu HY, Englund JA, Bedford T, Matsen FA, Boni MF, and Bloom JD (2024). “Age-dependent heterogeneity in the antigenic effects of mutations to influenza hemagglutinin”. Cell Host & Microbe 32.8, 1397–1411.e11. DOI: 10.1016/j.chom.2024.06.015. PMC11329357 39032493

[R63] Wilson JR, Guo Z, Tzeng WP, Garten RJ, Xiyan X, Blanchard EG, Blanchfield K, Stevens J, Katz JM, and York IA (2015). “Diverse antigenic site targeting of influenza hemagglutinin in the murine antibody recall response to A(H1N1)pdm09 virus”. Virology 485, pp. 252–262. DOI: 10.1016/j.virol.2015.08.004. PMC5737639 26318247

[R64] Wolf YI, Viboud C, Holmes EC, Koonin EV, and Lipman DJ (2006). “Long intervals of stasis punctuated by bursts of positive selection in the seasonal evolution of influenza A virus”. Biology Direct 1.1. DOI: 10.1186/1745-6150-1-34. PMC1647279 17067369

[R65] World Health Organization (2025). Recommended Composition of Influenza Virus Vaccines for Use in the 2026 Southern Hemisphere Influenza Season. Tech. rep. World Health Organization. https://www.who.int/publications/m/item/recommended-composition-of-influenza-virus-vaccines-for-use-in-the-2026-southern-hemisphere-influenza-season.

[R66] World Health Organization (2026). Recommended Composition of Influenza Virus Vaccines for Use in the 2026–2027 Northern Hemisphere Influenza Season. Tech. rep. World Health Organization. https://www.who.int/publications/m/item/recommended-composition-of-influenza-virus-vaccines-for-use-in-the-2026-2027-northern-hemisphere-influenza-season.

[R67] Wrammert J, Smith K, Miller J, Langley WA, Kokko K, Larsen C, Zheng NY, Mays I, Garman L, Helms C, James J, Air GM, Capra JD, Ahmed R, and Wilson PC (2008). “Rapid cloning of high-affinity human monoclonal antibodies against influenza virus”. Nature 453.7195, pp. 667–671. DOI: 10.1038/nature06890. PMC2515609 18449194

[R68] Yisimayi A, Song W, Wang J, Jian F, Yu Y, Chen X, Xu Y, Yang S, Niu X, Xiao T, Wang J, Zhao L, Sun H, An R, Zhang N, Wang Y, Wang P, Yu L, Lv Z, Gu Q, Shao F, Jin R, Shen Z, Xie XS, Wang Y, and Cao Y (2024). “Repeated Omicron exposures override ancestral SARS-CoV-2 immune imprinting”. Nature 625.7993, pp. 148–156. DOI: 10.1038/s41586-023-06753-7. PMC10764275 37993710

